# Investigating the Use of Diagnostic Genes in Integrated Monitoring with a Laboratory and Field Study on Flounder (*Platichthys flesus*)

**DOI:** 10.3390/toxics13030203

**Published:** 2025-03-12

**Authors:** Michelle C. Giltrap, Michael J. Leaver, Kelly White, James G. Wilson, Atiqur Rahman, Adrian Maguire, Aidan D. Meade, Janina Baršiene, Craig D. Robinson

**Affiliations:** 1 School of Food Science and Environmental Health, Technological University Dublin, City Campus, Grangegorman, D07 ADY7 Dublin, Ireland; 2 Radiation and Environmental Science Centre, Physical to Life Sciences Hub, Technological University Dublin, D08 CKP1 Dublin, Ireland; 3 Institute of Aquaculture, University of Stirling, Stirling FK9 4LA, UK; 4 Marine Laboratory, Marine Scotland, 375 Victoria Road, Aberdeen AB11 9DB, UK; 5 Zoology Department, Trinity College Dublin, College Green, D02 PN40 Dublin 2, Ireland; 6 Department of Agricultural Chemistry, Bangladesh Agricultural University, Mymensingh 2202, Bangladesh; 7 School of Physics, Clinical and Optometric Sciences, Technological University Dublin, City Campus, Grangegorman, D07 ADY7 Dublin, Ireland; 8 Nature Research Centre, 08412 Vilnius, Lithuania

**Keywords:** qPCR array, flounder, biomarker, contaminants, monitoring

## Abstract

For many years, there has been increasing concern about the effects of the presence of hazardous substances in the environment. The chemical and biological effect (BE) monitoring of these pollutants has proven difficult due to low environmental concentrations, variable bioavailability, and the generalised nature of ecological responses to these substances. The over- or under-expression of key genes has proven to be useful in understanding the molecular mechanisms of the toxicity of contaminants. This study uses a quantitative PCR array to detect the changes in gene expression in flounder livers after exposure to both laboratory- and field-based contaminants. The model contaminants included 17β-estradiol (E2), 3-methylcholanthrene (3-MC), a commercial mixture of polychlorinated biphenyls (PCB, Arochlor), perfluoroctanoic acid (PFOA), and lindane. Multivariate analysis was used to investigate relationships between higher-organisational-level biomarkers, supporting parameters, and genes. A scoring system enabled the visualisation of biological effect responses and contaminants in field samples. Although gene expression was useful for inferring the pathways of toxicity in this organism, we recommend that this array be used in combination with existing and recommended higher-level biomarkers and should not be used as a replacement for traditional biomarkers currently used in monitoring.

## 1. Introduction

There is a requirement for the ecological status of transitional and coastal water bodies to be monitored under the Water Framework Directive [2000/60/EC] [1]. The WFD requires monitoring and classification of all European surface and ground waters using several biological and physico-chemical criteria in order to ensure “Good Ecological Status” of all water bodies. This Directive provides an important legislative opportunity to promote and implement an integrated approach to the risk assessment of contaminant levels and biological effects. Transitional waters including estuaries can potentially be under environmental pressure due to natural and anthropogenic sources, presenting several monitoring difficulties. In addition to this and overlapping geographically with the WFD, the Marine Strategy Framework Directive [2008/56/EC] [2] aims to achieve and maintain “Good Environmental Status” (GES) in coastal waters. Under both of these directives, reliable tools are required to monitor environmental health and assess the biological effects of anthropogenic activities on aquatic organisms. Whilst it is clear that monitoring long-term effects at higher biological levels of organisation, such as populations and ecosystems, is important, responses at the molecular and cellular levels provide rapidly detectable and highly specific early warnings of a pollutant’s effect on the organism. In line with MSFD objectives, a robust set of tools are required to link the causes and effects of contaminants. There is an urgent need to develop and utilise new assays capable of evaluating both short- and long-term changes in organisms in relation to contaminants.

Estuarine and coastal environments can potentially be major receivers of contaminants from industrial and domestic inputs [3,4]. The European flounder (*Platichthys flesus* L.) resides in estuarine environments during its juvenile stage and being a benthic feeder in direct contact with sediments, this organism has the potential to accumulate contaminants. With its widespread distribution in both marine and brackish waters and presence in both clean and polluted estuaries, it is currently a recommended species for monitoring throughout many programmes [5]. Impacts such as disturbances of reproductive success [6], negative impacts on some flatfish species after short-term exposure to pharmaceuticals [7], increased disease prevalence [8], and high tumour frequencies [9] are a few examples of the effects on flounder that inhabit contaminated systems, making this a key sentinel species for the monitoring of pollution effects.

The Oslo and Paris Commission (OSPAR) and the International Council for Exploration of the Sea (ICES) recommend an integrated monitoring framework, whereby a toolkit of biological effects and contaminant measurements are applied and evaluated to sentinel species such as the European flounder [10]. This weight-of-evidence approach includes using a range of indices at different organisational levels, from ecological community changes to disruption of enzyme function, and the final indicator choice is a compromise between the lag/inertia of large systems in terms of the dose–response to contaminants and the realism/practicality of small-scale assays. Also, most of these techniques are incapable of linking specific contaminants with the measured causal effects and respond to multiple stressors, and therefore, individual contaminants and effects cannot be determined. Monitoring programmes have used the integrated monitoring framework with some advantages and disadvantages, with only one biomarker (imposex in marine gastropods) currently listed as mandatory for monitoring due to the unequivocal link between tributyltin and the imposex response. The number of available biomarkers with this level of specificity is small. In addition to this, the environmental concentrations, the variability in biological effects, bioavailability, and the generalised nature of ecological responses to these substances make it difficult to investigate links between the contaminant and its effect.

There is therefore a pressing need to develop and utilise assays capable of evaluating both acute and chronic toxic effects and ones which can infer the mechanistic links between specific contaminant exposure and biological effects/biomarkers. Omic techniques, and in particular ecotoxicogenomics, aim to understand and predict toxicity mechanisms to understand the modes of action in response to exposure to a single or mixture of chemicals [11], with the most common techniques being quantitative PCR, microarrays, and, more recently, RNA sequencing [12]. Investigating gene expression using omics techniques after exposure to contaminants has been widely used for many fish, including zebrafish embryos [13], hornyhead turbot [14], largemouth bass [15], and flounder [16,17], and these have also been applied in field studies [18], mesocosms [19] and laboratory exposures [20]. More recently, gene expression has been used to infer pathways that can lead to cancer in flatfish [21]. However, it is difficult to unequivocally link these pathways to pollutant exposure/cancer due to the natural variability in parameters such as salinity, temperature, and feeding [19]. Fernandez-López (2024) [22] also reported the effects of salinity on fatty acid composition in flatfish, indicating changes due to natural stress in the environment. Leaver et al. (2010) [19] demonstrated that flounder, experimentally exposed long-term to polluted sediment, did not show the expected differences in diagnostic biomarker genes such as CYP1A that might have been expected upon exposure to PCB/PAH-contaminated sediment. The authors did suggest that other genes, such as the proapoptotic diablo gene, showed a pattern of expression dependent on the sediment origin, and the level of this gene was higher in all of the polluted samples compared to clean sediments. The overall outcomes of these omic experiments have suggested a subset of diagnostic genes which might be informative in biomonitoring scenarios and which could be used to develop a more practical qPCR array for contaminant biomonitoring. Furthermore, appropriate statistical models and procedures have been developed during these omic projects, which can help to discriminate populations of fish in different exposure scenarios [17].

This study includes a genetic analysis of biobanked tissues from a previous study [23] and maximises the information gained from these historic experiments. The samples collected during the previous study are used to compare qPCR model compounds to field monitoring data in the present study. The main objectives are to (1) develop a qPCR array using selected diagnostic genes in flounder. Genes were chosen on the basis of the available literature and recommended biomarker responses currently utilised in monitoring programmes (e.g. metallothionein protein) and those that have the potential to demonstrate exposure to model environmental contaminants. The other goals are to (2) evaluate the up/down regulation of genes after exposure to contaminants as a set of diagnostic gene ‘tools’ for future monitoring; (3) determine hepatic expression in wild-caught flounder from different locations; and (4) using a scoring system and starplots, investigate if visual relationships between gene expression, biomarker, and contaminant measurement data can be demonstrated; and (5) explore site differences with multivariate analysis in relation to contaminant exposure, gene expression, and biomarkers.

## 2. Materials and Methods

### 2.1. In Vivo Fish Exposure

The samples of laboratory-exposed fish used in this study were collected as described in a previous study [23], whereby a range of model contaminants were administered via intraperitoneal injection (i.p) into flounder and sampled sequentially over a 16-day exposure. The flounder samples used for this present study were collected 4 days after treatment. For these i.p. treatments, *P. flesus* were artificially reared from gametes obtained from three females and three males and maintained on a commercial pelleted trout diet in a flowing seawater aquarium system (8 °C, salinity 32 ppt, 60 light/40 dark) at Port Erin Marine Laboratory, Isle of Man, UK. At 2–3 months post-hatch, juveniles were transferred to a recirculating sea water system at Stirling University, Scotland and were grown at 11 °C and maintaining a salinity of 32 ppt. Sexually immature (macroscopically undifferentiated or male) fish of 75–100 g were treated by intraperitoneal injection, 3-methylcholanthrene (3-MC, a planar polycyclic aromatic hydrocarbon, 25 mg/kg in olive oil), Aroclor 1254 (a mixture of polychlorinated biphenyl (PCB) congeners, 50 mg/kg in olive oil), lindane (g-hexachlorocyclohexane, an organochlorine pesticide, 25 mg/kg in olive oil), perfluoro-octanoic acid (PFOA, a persistent pollutant and peroxisome proliferator, 100 mg/kg in olive oil), olive oil (1 mL/kg), or saline (0.9%). All exposure experiments were carried out in compliance with UK regulations governing animal experimentation (UK Home Office licenses PPL 60/2360 and PIL 60/3279) in aerated static sea water tanks (80 cm × 80 cm × 40 cm containing 160 L water) sited in a constant-temperature containment aquarium. Water was replaced every 2 days over the 16-day time period. After 4 days, groups of at least 5 fish sacrificed by a blow to the head and sampled. Fish length, body weight, liver, kidney and gonad weights were recorded, and samples of liver tissue were excised and stored at −80 °C until further analysis. Condition factors (K) were calculated by the formula (body weight/length^3^) × 100, liver somatic indices (LSIs) by (liver weight/body weight) × 100, and gonad somatic indices (GSIs) by (gonad weight/body weight) × 100, but these data are not reported. All male fish used in this study were immature males. Female fish exposure data are available but not shown.

### 2.2. Sampling and Survey Information for Field Samples

**Figure 1:**
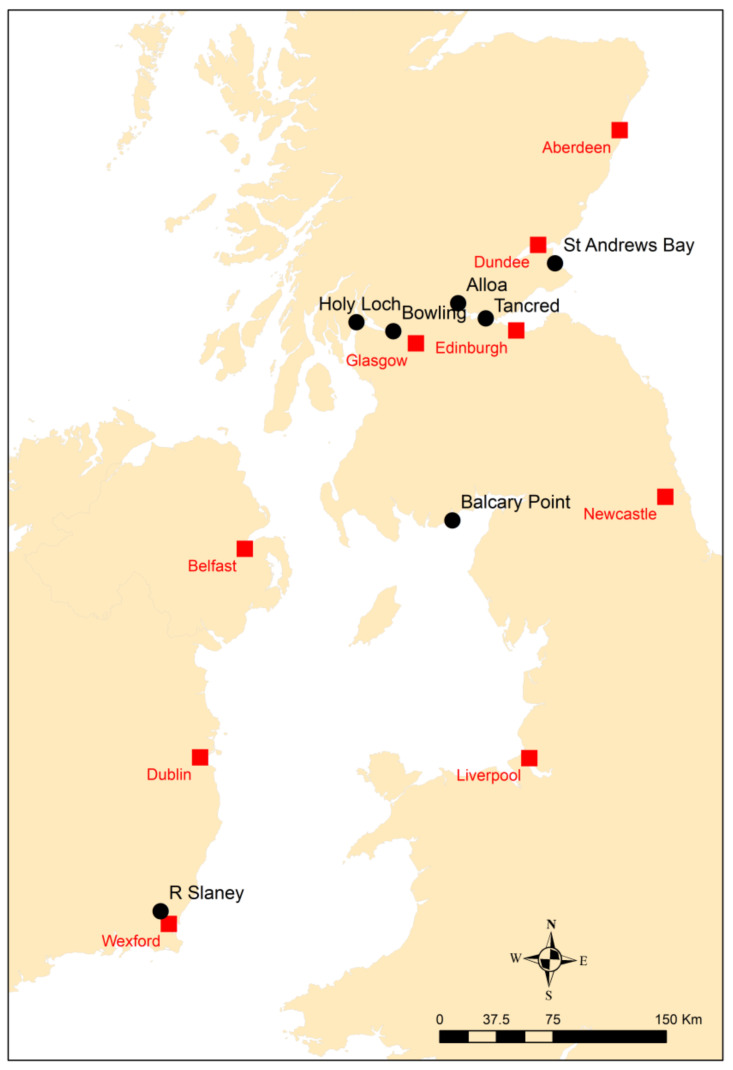
Map of sampling locations.

*P. flesus* (20–25 cm) were collected and sampled (following OSPAR guidelines) on a number of surveys between 2009 and 2010. A map of the sampling areas is shown in Figure 1. The Scottish research vessel *Alba na Mara* was used to obtain flounder in November 2009 and November 2010. In 2009, fish were obtained from Balcary Point (Solway Firth) and off Holy Loch (inner Firth of Clyde) using a bottom trawl fitted with 50 mm cod end and towed at 2.5 kt for 60 min. In 2010, fish were similarly collected from St Andrews Bay (Scottish east coast) and again at Holy Loch, whilst at Bowling (Clyde estuary), Alloa (upper Forth estuary), and Tancred Bank (lower Forth estuary), flounder were collected using a 2 m beam trawl fitted with a 50 mm cod end and towed for 20–30 min at 2.5 kt. Repeated tows were undertaken at each location until the required number of fish were obtained. Fyke nets were also used to obtain fish. Fish sampled from the Slaney estuary in Ireland using a small boat and seine net and were immature (size range 15–20 cm).

Once aboard the Scottish research vessel, fish were kept alive in tanks containing fresh running seawater. All fish above 20 cm were chosen for sampling and dissections were performed within 2 h of capture. Fish size (length and weight), gender, and external diseases were recorded. Contamination was minimised between sites by flushing tanks and water change between locations. The fish were sacrificed by a blow to the head and severance of the spinal cord from the brain, and immediately blood was sampled for plasma vitellogenin (Vtg) and micronucleus (MN) analysis with heparin-coated syringes. Blood for MN was smeared on microscope slides, fixed in methanol, and allowed to air dry. Blood for Vtg was centrifuged at 3000× *g* and the plasma removed and snap-frozen in liquid nitrogen. Bile was collected from the gall bladder and stored at −20° C. Livers were excised and aliquots separated for qPCR and 7-ethoxyresorufin (EROD) assay. Liver samples for qPCR were placed in RNA later and snap-frozen in liquid nitrogen. Livers for EROD were homogenised in phosphate buffer containing dithiothreitol and EDTA, centrifuged at 10,000× *g* and the S9 fraction removed and snap-frozen in liquid nitrogen (Scotland), or tissues were snap-frozen in liquid nitrogen and subsequently homogenised upon return to the laboratory (Ireland). Pooled flesh samples (five pools per site, each of 5–7 fish) and remaining liver tissues (five pools of 5–7 per site) were sampled for contaminant determinations and otoliths were sampled for aging. Tissue for biomarker work was immediately placed in liquid nitrogen on board and subsequently transferred to the −80 °C freezer until analysis.

### 2.3. Determination of Contaminant Concentrations and Biological Effects in Field-Caught Fish

Samples for contaminant analysis were pooled in groups of 5 mixed-gender fish. Trace metals were determined in fish liver and muscle samples by microwave digestion (Multiwave 3000, Anton Paar, Luton, UK) and inductively coupled plasma mass spectrometry (Elan 6100*DRC+* with AS-90/91 autosampler, Perkin-Elmer Sciex, Beaconsfield, UK), as previously described [24,25]. Samples of livers for contaminant analysis were pooled in groups of 5 mixed-gender fish and trace metals, polychlorobiphenyls (PCBs), and polybrominated diphenyl ethers (PBDEs) were determined in fish liver by accelerated solvent extraction and gas chromatography mass spectrometry [26,27]. Hepatic EROD activity was determined in S9 post-mitochondrial fractions using a Perkin Elmer LS55 fluorometer [28,29] and normalised for protein content using the Lowry (1951) method [30] with bovine serum albumin as standard. Bile samples were analysed for PAH metabolites using synchronous fluorescent spectroscopy (with a 42 nm difference in excitation and emission wavelengths) and standard addition quantification using 1-hydroxypyrene, modified from Ariese et al., 2005 [31]. Micronuclei were determined [32] on 4000 erythrocytes per fish following Giemsa staining. Vtg was determined by ELISA [33]. Determination of contaminants and of EROD activity were accredited to ISO17025 [34] by the UK Accreditation Service.

### 2.4. RNA Extraction and cDNA Synthesis for Laboratory and Field-Exposed Fish

RNA extraction was performed using an organic extraction protocol. Tri reagent (1 mL) was added to 100 mg liver tissue in small pieces on ice (20 min). Samples were homogenised in a bead beater for 60 s. Homogenised samples were incubated at room temperature (RT) for 5 min followed by centrifugation at 12,000× *g* for 10 min at 4 °C. Moreover, 1-bromo-3-chloropropane (BCP) was added (100 μL per ml of tri reagent) and shaken for 15 s. Samples were then incubated for 15 min at RT followed by centrifugation at 20,000× *g* for 15 min at 4 °C and the aqueous layer removed for further analysis. RNA was precipitated using half volume (per aqueous phase volume) RNA precipitation solution (1.2 M sodium chloride and 0.8 M sodium citrate sesquihydrate) and half volume of isopropanol followed by incubation for 10 min at RT. This was followed by centrifugation at 20,000× *g* for 10 min at 4 °C. The RNA pellet was washed with 1 mL of 75% ethanol for 15 min at RT and centrifuged at 20,000× *g* for 5 min at RT and cautiously removing all traces of ethanol by air-drying for 5 min. RNA was then dissolved by re-suspending the pellet in 100 µL of RNase-free water and samples were incubated for 60 min with gentle inverting to aid resuspension, and final RNA concentrations and purity were measured using a Nanodrop 1000 spectrophotometer. RNA integrity was checked with gel electrophoresis using a 1% agarose gel.

Complementary DNA (cDNA) was synthesized from total RNA. Each reaction of 20 μL contained 2 μg of total RNA, 4 μL 5× buffer, 1 μL dNTP mix (10 mM each), 1 μL random primers, 1 μL oligodT, 1 μL Superscript II (Invitrogen, Thermo Fisher Scientific, Loughborough, UK), 2 μL 100 mM DTT and 8 μL ultrapure water. cDNA synthesis was performed in a PCR machine (Tprofessional, Biometra Thistle Scientific, Glasgow, UK) at 42 °C for 1 h and stopped by 70 °C for 15 min. cDNA was diluted to 200 μL with ultrapure water and stored at −20 °C until further analysis.

### 2.5. Quantitative PCR (q-PCR)

q-PCR was performed using a Toptical Biometra Thistle Scientific, Glasgow, UK. Quantitative PCR for each gene was performed in a total volume of 16 μL containing 5 μL cDNA, 100 nM of each primer and 10 μL of SYBR green QPCR mastermix (LightCycler^®^ 480 SYBR Green I Master, Roche Diagnostics, Burgess Hill, UK). For each of the target genes, forward and reverse primers were chosen from the available EST sequences by using Primer-BLAST “http://www.ncbi.nlm.nih.gov/tools/primer-blast” (accessed on 20 February 2015). Target genes were selected based on previous studies of flounder that identified hepatic mRNAs which varied in expression level depending on pollutant status of sediments (Table 1; [17,19]). Genes selected included those with functions or are markers in xenobiotic metabolism, stress response, apoptosis, and endocrine disruption. Primer pairs and database information are provided in Supplementary Table S1. Thermal cycling was initiated with incubation at 95 °C for 15 min in order to activate the DNA polymerase present in the mix. After this initial step, forty-five cycles of PCR were performed. Each PCR cycle consisted of heating for 15s at 95 °C for denaturing, and then for 15 s at 60 °C and 30s at 72 °C for annealing and extension. Cycle threshold (C_T_) values corresponded to the number of cycles at which the fluorescence emission, which was monitored in real time, exceeded the threshold limit. Melting curve analysis was performed to indicate the production of a single product in each reaction. For each individual fish, all target and reference genes were assayed in triplicate cDNA samples on the same 96-well assay plate.

### 2.6. Gene Expression

The genes selected for this study and their functions are described in Table 1. Relative changes in gene expression were calculated according to the Pflaffl method [35], and individual specific primer efficiency was calculated by linear regression of flourescence versus logCt values from serial dilutions of flounder cDNA. The calculated relative expression ratio of each gene was based on the PCR efficiency (E) and C_T_ of the samples compared with control and expressed in comparison to the reference genes i.e. α-tubulin (ATUB) and elongation factor 1 (EF1).

### 2.7. Assessment Procedure and Scoring Index

Assessment criteria are derived from the ICES guidelines on integrated monitoring and the OSPAR coordinated environmental monitoring programme [10,36], where background concentrations (BCs), background assessment concentrations (BACs), and environmental assessment criteria (EACs) for contaminants in biota are set. Species-specific assessment criteria for EROD, PAH bile metabolites, Vtg and MN as per [10] were used for assessment. For biomarker data, the mean + 95% confidence interval was compared. Contaminant assessment criteria in fish were applied as per [36]. For contaminant data, lipid-normalised values were used for comparison to assessment criteria.

An index based on a method used by Giltrap et al. (2017) [37] was used for quantitatively comparing the biomarker, contaminant, and gene scores. The index was based on a method by [38] and adapted with the following modifications. Only contaminants where AC were available were used for assessment, as per Table 2. Only scores were derived for available contaminant data in liver tissue. A score for each contaminant at each site was calculated by how many times it exceeded the EAC. The parameter which exceeded the EAC to the greatest extent was designated a score of “10”, with other site/sample scores assigned a pro-rata score relative to the highest one. Finally, a score was created for each individual PCB and group of PCBs, and an overall score for the sample/site was obtained by using a method described by [38], as follows: [(B1 × B2)/2] + [(B2 × B3)/2] + …[(Bn − 1 × Bn)/2] + [(Bn × B1)/2]}. B1, B2, …Bn are the derived scores for the contaminants and divided by n parameters. In order to generate a score for biomarker data, this method was repeated. Star plots were then created in Excel in order to visualise the data.

### 2.8. Statistical Analyses

Lists of differentially expressed genes were generated by finding genes that differed by more than two-fold in normalised data and demonstrated statistically significant differences between exposed and control groups. This was tested using the Wilcoxon rank sum test in RStudio version 1.0.153.

To produce a model summarising the gene subsets differentiated between exposure conditions and exposure sites, linear discriminant analysis (LDA) was used. This is described thoroughly elsewhere [40]. Briefly, LDA produces a model which uses linear combinations of variables to maximise the between-class variance (where in the current instance, class may be viewed in terms of location or exposure condition) whilst minimising the within-class variance. LDA was performed on gene expression, biomarker data, and contaminants (metals, PCBs and PBDEs). In the present work, LDA was used to generate models that allow the visualisation of the discrimination between exposure condition or location and the interpretation of the origin of this discrimination in terms of the loadings on the original variables within each LD. In order to investigate links between biomarkers and gene expression, multivariate regression analysis was performed in R version 4.4.2 (31 October 2024)—“Pile of Leaves”.

## 3. Results

### 3.1. Fish Exposure and Diagnostic Genes

In response to all treatments, quantification of gene response profiles by the cDNA array over the period of exposure (4 days) showed clear and specific changes in gene expression in liver tissue. 

**Figure 2:**
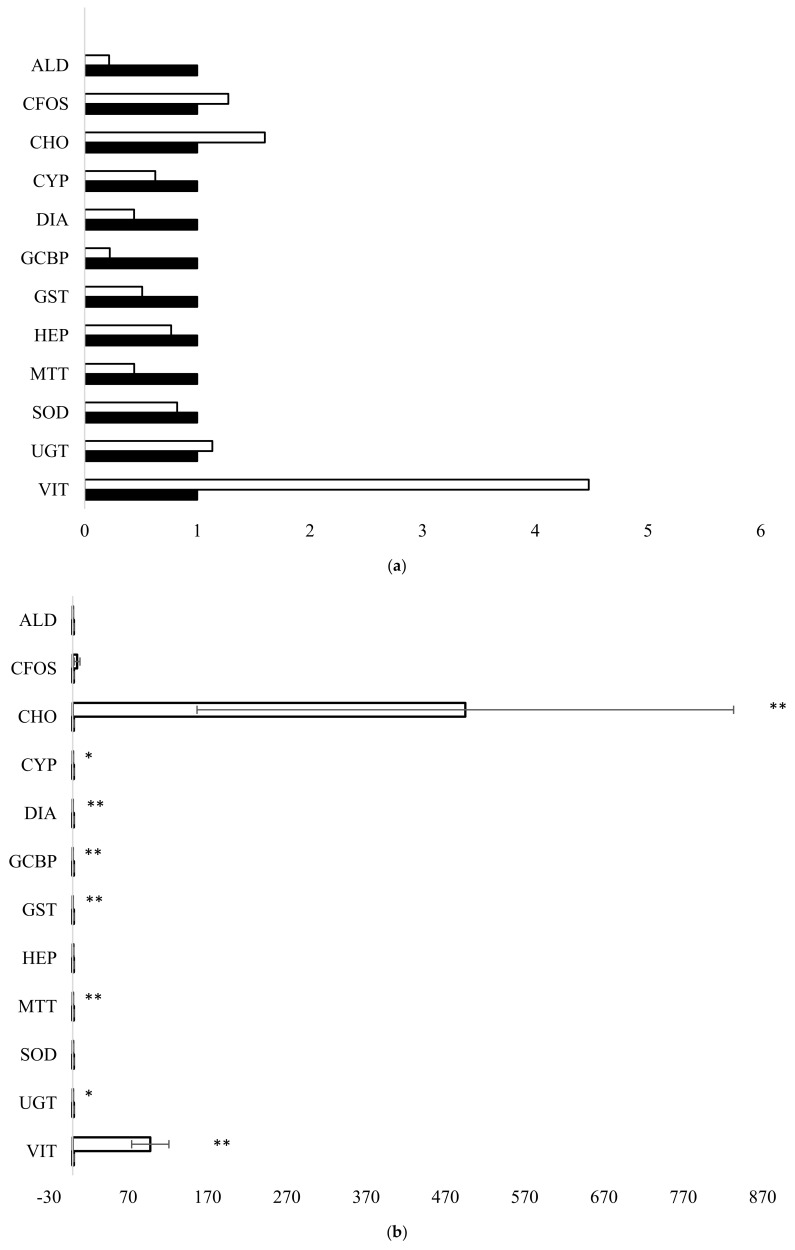

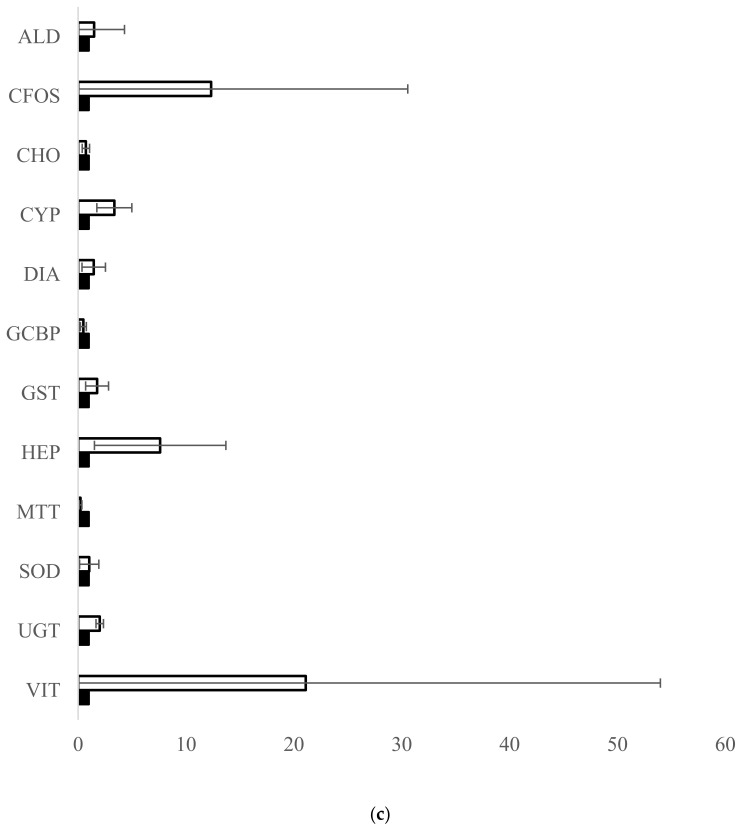

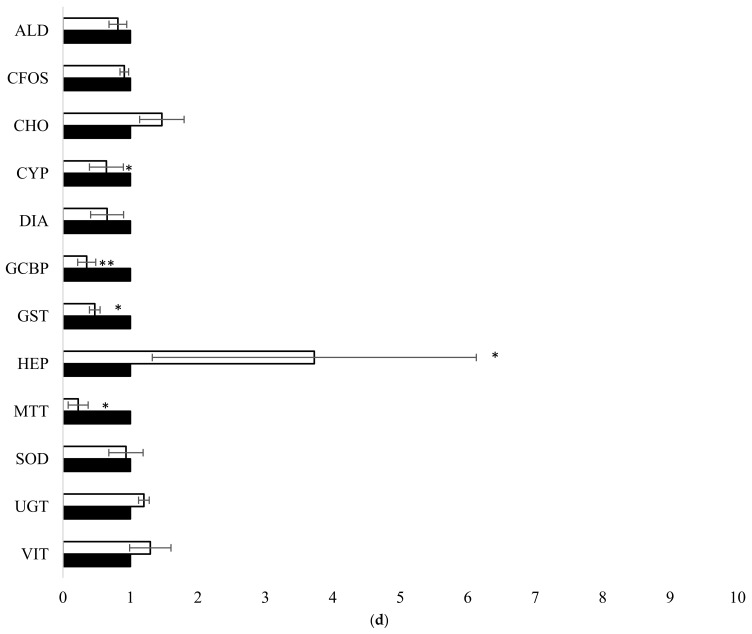

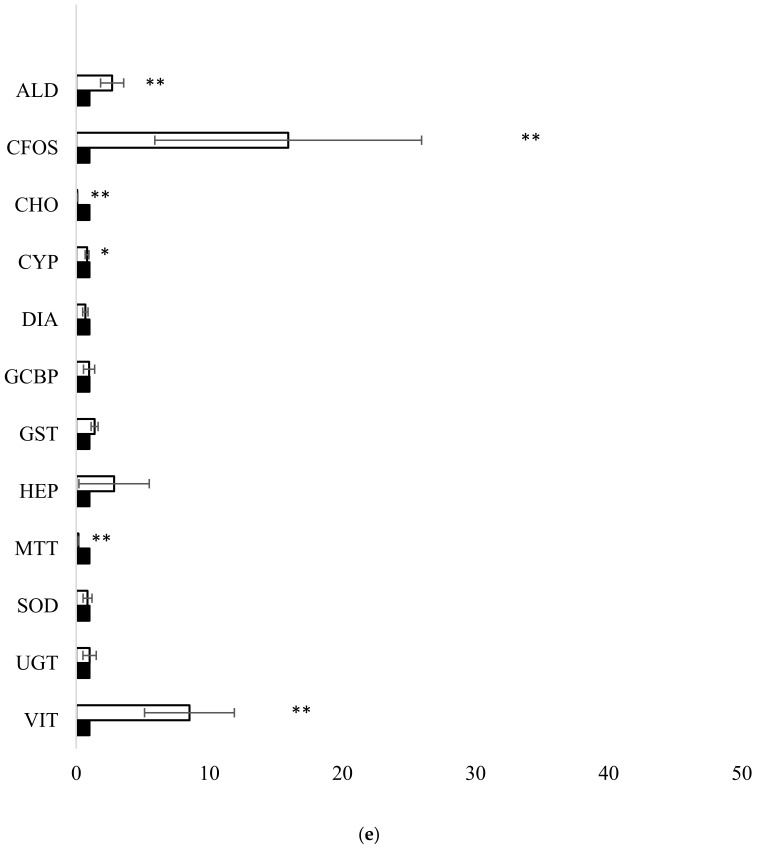
(**a**) Gene expression in control [∎] and olive oil-exposed (injected) [⧠] mature male flounder (*n* = 3) with significant differences to unexposed saline control indicated. (**b**) Gene expression in control [∎] and 17β-estradiol-exposed [⧠] mature male flounder (*n* = 4) with significant differences to unexposed control indicated. (**c**) Gene expression in control [∎] and 3-MC cholanthrene-exposed [⧠] immature male flounder (*n* = 3) with significant differences to unexposed saline control indicated. (**d**) Gene expression in control [∎] and lindane-exposed [⧠] mature (*n* = 2) and immature (n = 2) male flounder with significant differences to unexposed saline control indicated. (**e**) Gene expression in control [∎] and PFOA-exposed [⧠] immature male flounder (*n* = 4) with significant differences to unexposed saline control indicated. *p* < 0.01 = **, *p* < 0.05 = *.

Following intraperitoneal injection in male fish, differences in gene expression were observed in the carrier olive oil and model contaminant treatments (Figure 2a–e).

Overall, model contaminant exposures resulted in some significant gene expression changes. In olive oil-injected male fish, none of the genes were significantly up- or down-regulated. For E2 exposed male fish, VIT (*p* < 0.01), CHO (*p* < 0.01), and CFOS genes were significantly induced in male fish, while UGT (*p* < 0.05), MTT (*p* < 0.01), GST (*p* < 0.01), GCBP (*p* < 0.01), DIA (*p* < 0.01), and CYP (*p* < 0.05) were down-regulated. For E2-exposed male fish, there was 50- and 10-fold up-regulation of the CHO and VIT genes, respectively, compared to the olive oil controls. In 3-MC-exposed fish, VIT, HEP, DIA, CYP, and C-FOS were up-regulated in exposed male fish, while MTT was down-regulated; however, this was not statistically significant. In lindane-exposed fish, HEP was significantly (*p* < 0.01) up-regulated, while MTT (*p* < 0.05), GCBP (*p* < 0.01) and CYP (*p* < 0.05) were down-regulated. In the PFOA-exposed fish, VIT (*p* < 0.01), CFOS (*p* < 0.01), and ALD (*p* < 0.01) were significantly up-regulated, while MTT (*p* < 0.01), CYP (*p* < 0.05), and CHO (*p* < 0.01) were significantly down-regulated. The hepcidin gene was also up-regulated, but this was not significant. Overall, there were significant acute effects demonstrated in male flounder at the gene level after a short exposure time of 4 days to model contaminants. 

### 3.2. Contaminants in Wild-Caught Fish

All assessment criteria used in this study are outlined in Table 2. Contaminant concentrations in wild-caught fish including metals, PCBs, and PBDEs are presented in Table 3. Where assessment criteria were available, metal and PCB concentrations were compared and classified as per [36] (i.e., blue (<BAC), green (>BAC <EAC), and red (>EAC)) and are presented in Table 3.

Overall, when comparing contaminant values against assessment criteria for metals and PCBs, most sites demonstrated moderate levels of contaminants with many values below the EAC. An exception to this was demonstrated in flounder sampled from Holy Loch in 2009, which showed levels of some PCBs (CB28, CB52 and CB118) above the EAC (Table 3). When comparing the biomarker data against available assessment criteria, moderate levels of effects were observed, with most responses below the EAC but above the BAC. There was a clear overall induction/deduction of some gene responses in flounder from contaminated sites (Supplementary Figure S1a–g). The significant induction of VIT and CHO genes in fish from Holy Loch in 2009 (Supplementary Figure S1b) could potentially indicate exposure to estrogenic contaminant(s) at this site. 

### 3.3. Biomarkers and Supporting Parameters in Wild-Caught Fish

The biomarker data including EROD, bile metabolites, Vtg, and MN are presented in Table 4 and compared to the criteria as per [10] (i.e., response <BAC (blue) and >BAC <EAC (green), >EAC (red)). None of the biomarker responses exceeded the EAC. For Vtg measurements, the limit of quantification (0.2 µg mL^−1^) was not sufficient to compare the assessment criteria for BAC in male flounder (0.13 µg mL^−1^), and currently there are no EACs available for male flounder. Levels of micronuclei in flounder were two times higher than the BAC in the fish from Balcary Point and were shown to be above the BAC from other locations. Bile metabolite concentrations were above the BAC for most locations, with the exception of Balcary point and St. Andrews.

### 3.4. Multivariate Analysis of Contaminants in Flounder

Linear discriminant analysis (LDA) was used to investigate multivariate effects of metals in fish between locations (Supplementary Figure S2a). The clustering of LDA scores revealed differences in metals between locations where LD1 accounted for 66% and LD2 for 20% of the data. Alloa and Tancred clustered on the basis of LD1 rather than LD2, with lower values of Cd, Pb, Fe, and Cu and higher values of selenium, mercury, and vanadium. All other clusters were discriminated using LD2, representing an increase in mercury and a decrease in cadmium and copper. Flounder from St. Andrews had higher silver, cadmium, arsenic, and copper and lower mercury, nickel, and lead compared with the other locations (LD1). Spatial differentiation between locations due to particular metals is evident. Multivariate effects of PCBs at different locations proved difficult with low sample numbers for some areas, but in general, the locations separated well with LDA (Supplementary Figure S2b). Holy Loch (both years) clustered on the basis of LD2 rather than LD1. All other clusters were discriminated using LD1. Differences in Holy Loch data are due to variation of PCBs on the basis of CB153 and CB97 (positive scores) and CB49 and CB180 (negative scores). An LDA analysis of BDEs at different locations is presented in Supplementary Figure S2c. Negative scores for LD1 are a result of increases in BDE66 and decreases in BDE100 and BDE154. Positive scores demonstrated that there were lower BDE66 and higher BDE100 and BDE154 in Alloa, Holy Loch, and Bowling flounder compared with the other locations, with Holy Loch showing the most extreme difference in terms of the BDEs.

### 3.5. Multivariate Analysis of Biological Effects in Wild-Caught Fish

Site differences were investigated with the biological effects (EROD, bile and MN) and supporting parameter data (GSI, LSI and condition factor (K)). LDA was performed on BE data and supporting parameters in both male and female wild-caught fish. There were no significant differences in clustering between male fish; however, female fish at Balcary Point clustered on the basis of LD1 scores, which represents an increase in K, GSI, LSI and EROD. Correlations were evident between age, weight and length between locations and, in general, fish were older at Balcary Point than elsewhere. In order to investigate relationships between gene expression profiles and higher-organisational-level biomarkers (EROD, bile, and MN), multivariate regression was used to predict whether there was a relationship between gene expression profiles and biomarkers. For all three biomarkers (EROD, MN, and bile metabolites), it was found that none of the genes could predict the biomarker response. However, there was potentially an association between EROD and the SOD gene, with a marginal significance of *p* < 0.05.

### 3.6. Biomarkers, Contaminants, and Gene Expression in Wild-Caught Fish

Since the data on contaminant level were found for mixed-gender samples (*n* = 5), it was difficult to predict whether the biomarker response or gene expression could be correlated to any of the measured contaminants with statistical differences clearly evident between individual male and female biomarkers and gene expression in each location. For this reason, a Starplot visualisation approach was taken in order to visually establish links between biomarkers with a specific mode of action (MOA) (e.g., receptor-mediated induction of cytochrome P450-dependant monooxygenases, specifically the CYP1A subfamily), oxidative stress, and endocrine disruption. Contaminants from the literature that could be associated with these MOA pathways were also visualised with this approach.

### 3.7. Scoring and Star Plots

**Figure 3:**
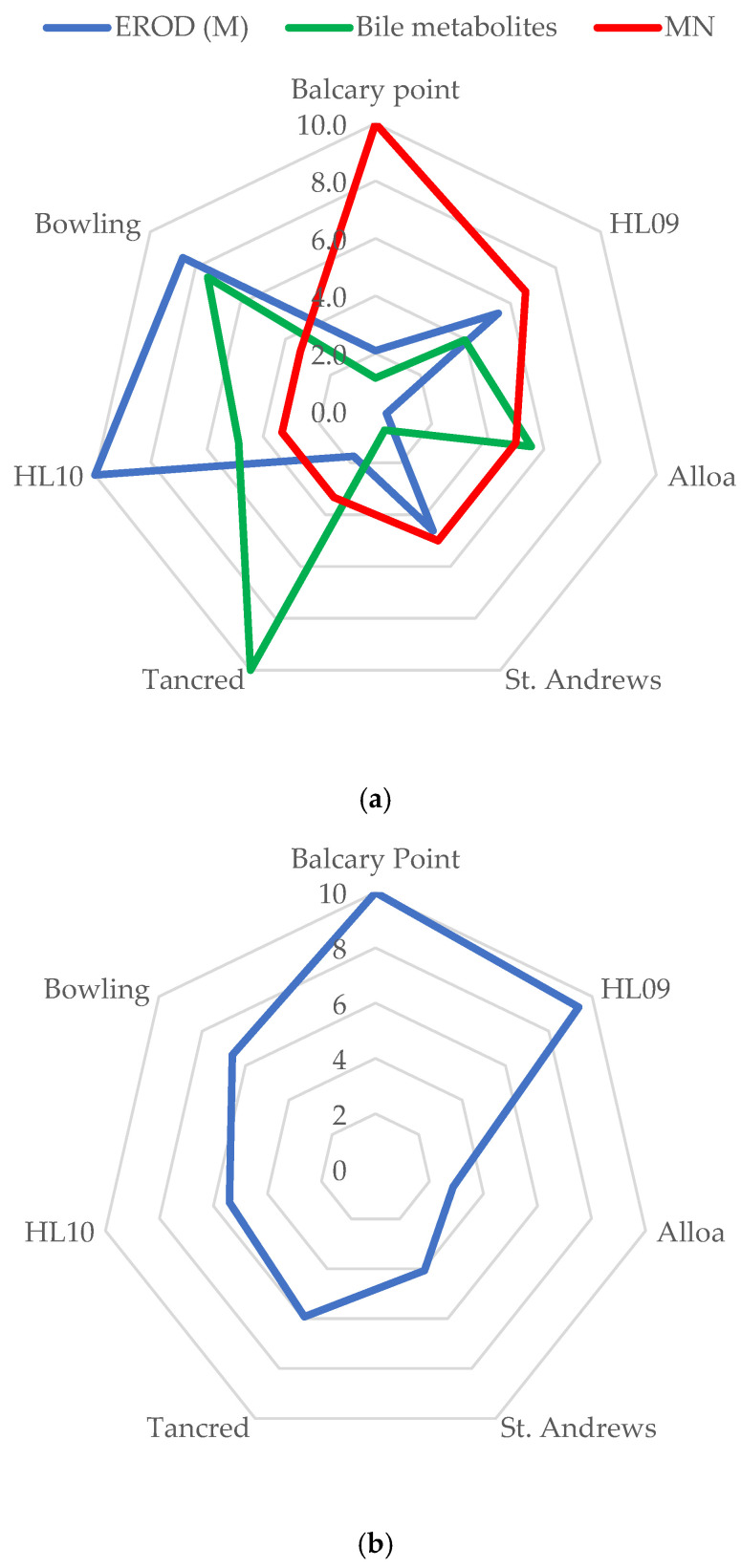

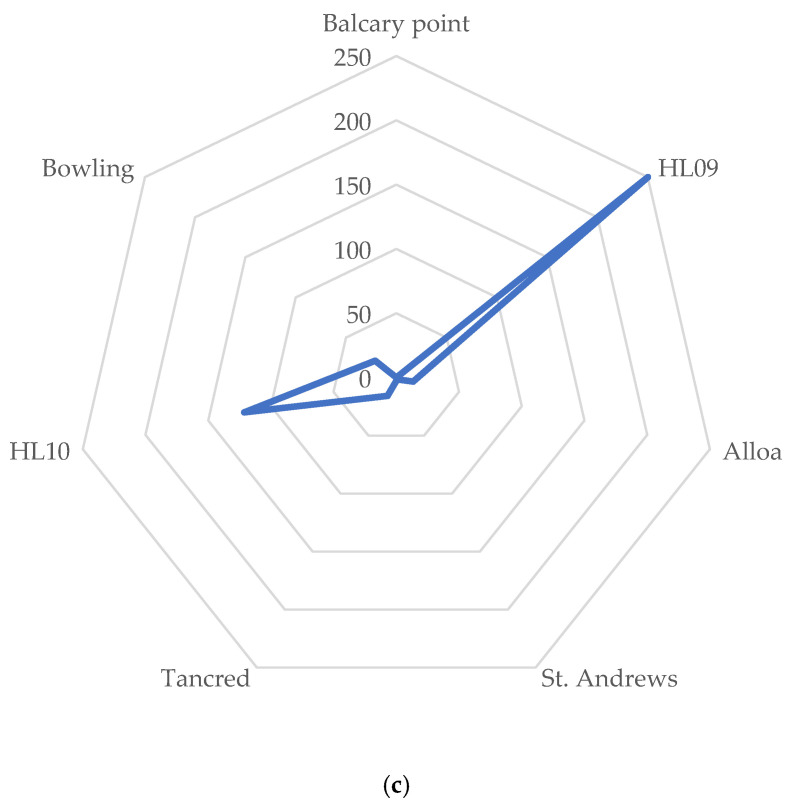
(**a**) Starplots showing scores of EROD, bile metabolites, and micronuclei in male flounder at each location. (**b**) Starplots of scores of CYP gene in male flounder. (**c**) Starplots of scores of PCBs in male flounder at each location.

**Figure 4:**
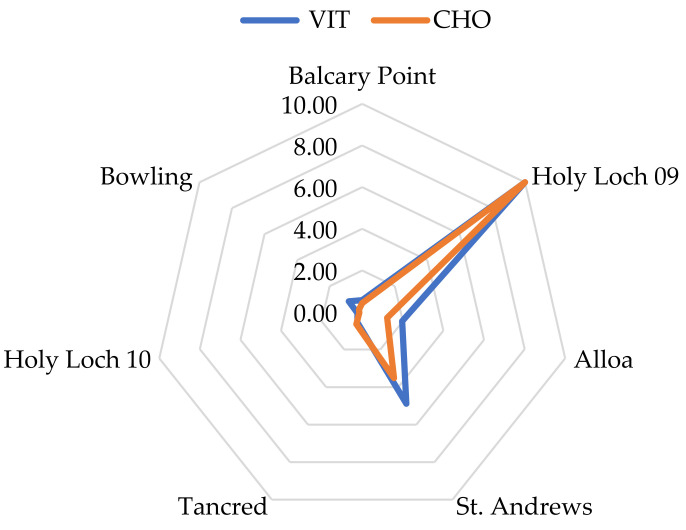
Starplots of scores of VIT and CHO in male flounder at each location.

**Figure 5:**
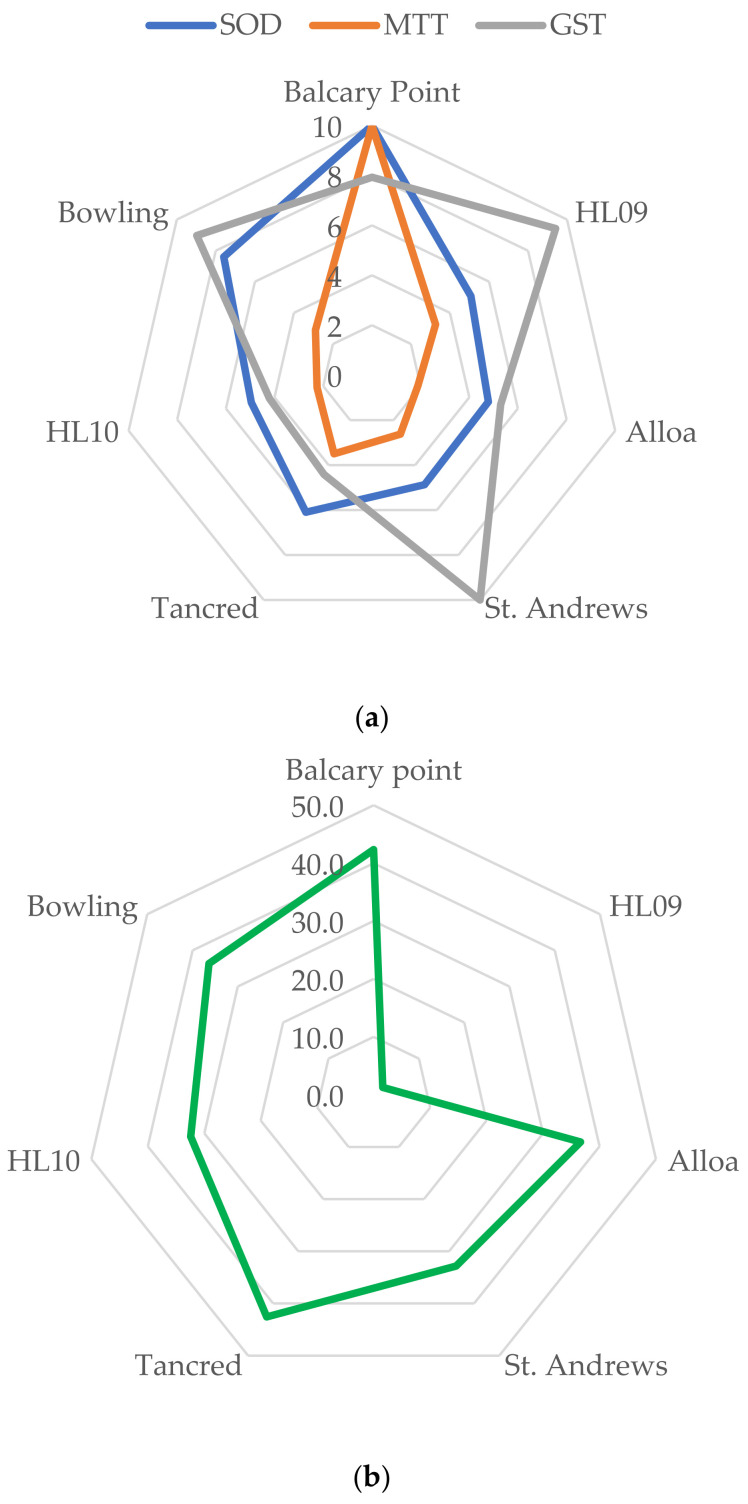
(**a**) Starplots of scores of SOD, MTT, and GST genes in male flounder. (**b**) Starplot of scores of total metals in male flounder at each location.

Where assessment criteria were available, scores were derived, and Starplots were produced for the biomarkers including EROD, bile metabolites, and MN (Figure 3a); Starplots were also produced for the CYP1A gene (Figure 3b) and PCB contaminant scores (Figure 3c). PCBs were clearly elevated at the Holy Loch site in both 2009 and 2010. There was a wide variation in up- and down-regulated genes in the wild-caught fish from different locations, and this is visualised with the scoring system and Starplots in Figure 3b (CYP1A), Figure 4 (VIT and CHO), and Figure 5a (SOD, MTT, GST) at each of the sites. As shown in Figure 3b, the CYP gene did not follow the same pattern as EROD at the sites as would have been expected, but it did follow a similar pattern to MN. Genes indicating potential exposure to endocrine-disrupting contaminants (VIT and CHO) were elevated at Holy Loch and St. Andrews compared with other locations (Figure 4). Figure 5a,b demonstrate oxidative stress-related genes (SOD, MTT and GST) and metal Starplots, respectively. With the exception of the Holy Loch site in 2009, the SOD gene followed a similar pattern to metals, indicating a potential link.

## 4. Discussion

### 4.1. Fish Exposure

Vitellogenin protein in male fish plasma is a well-known biomarker of estrogenic contamination [41] and has been used in environmental monitoring programmes for investigation of effects of estrogenic contamination in male fish [42,43]. For E2-injected fish in the present study, there was a clear and significant up-regulation of both the CHO and VIT genes. These two genes are well-known indicators of estrogenic compound exposure and have previously been shown to be up-regulated after 16 days of exposure to E2 in *P. flesus* in the study by Williams et al. (2007) [20]. A comparably lower level of induction of VIT and CHO was induced in some of the other model contaminant exposures, indicating greater sensitivity to E2 above other model contaminant exposures. This was in line with what was previously reported in [23], where some degree of induction of VIT and CHO occurred in all exposures after a given time.

The induction of hepatic cytochrome P4501A (CYP1A) is involved in the response to contaminants such as polycyclic aromatic hydrocarbons (PAHs) and planar PCBs, with induction of the gene being dependent on the aryl hydrocarbon receptor pathway [44]. Williams et al. (2008) [23] reported induction of CYP1A in the olive oil-injected fish after 16 days of exposure; however, in the present study, CYP1A was decreased in the olive oil controls after 4 days of exposure. There was a slight induction of a CYP1A response in the 3-MC-injected fish, although a lack of response to 3-MC in flounder has been noted previously [23]; notably, other PAHs, such as benzo[*a*]pyrene, and PCBs have been shown to strongly induce CYPIA in flounder [19]. Hepcidin was induced (but not significantly) in the 3-MC-exposed fish. This is a type II protein induced in response to inflammatory stimuli in fish [45,46] and was also induced in the lindane- and PFOA-injected fish in the present study. c-FOS was also induced in the 3-MC exposures, as well as VIT and ALD. PFOA is a persistent legacy pollutant and peroxisome proliferator which may act via PPAR-α and PPAR-γ in flatfish [14]. In the PFOA-injected fish, c-FOS and hepcidin were induced in the present study. Williams et al. (2008) [23] reported flounder to elicit strong stress and oxidative stress responses after exposure to PFOA. Hepcidin was induced in both the study by Williams et al. (2008) [23] and the present study. Overall, there is a clear cause-and-effect relationship demonstrated between fish injected with E2, which only showed induction of VIT and CHO genes.

### 4.2. Wild-Caught Fish

The CYP1A gene has a clear function in the metabolism of xenobiotic compounds such as PAHs and planar PCBs and was clearly induced in female fish from Holy Loch 2009 and 2010, which could possibly be a result of potentially higher levels of exposure to PCBs, which exceeded the EAC in flounder liver from this site. From the Starplots, Balcary also scored high for the CYP gene, but without the data for PCBs at this site, it is difficult to evaluate levels of potential exposure. Furthermore, EROD, the enzyme activity encoded by CYP1A, was found to be above BAC in male fish from Holy Loch in 2009 and 2010, and CYP1A transcripts were increased in Holy Loch 2009, although there was no evidence of this in male fish in 2010. The phase II detoxification gene, GST, was also up-regulated in female flounder from Holy Loch in 2009, whilst DIA, c-FOS, and ALD expression were suppressed. The up-regulation of the genes at the Holy Loch location and increased levels of contaminants and biomarker response found at this site indicate that the fish were responding to contaminants at the subcellular and organism level; however, it is difficult to make associations between biomarkers and contaminants, since the fish were separated by gender for biomarkers but not separated for contaminant analysis. There were plasma Vtg data available for flounder sampled from Holy Loch in 2010, with two male fish having elevated plasma vitellogenin concentrations (69.8 and 0.433 µg mL^−1^); that said, overall, male fish at this site showed suppressed VIT and CHO gene transcripts. Also, moderate levels of contaminants were demonstrated in fish livers from Holy Loch in 2010, with only PCB 118 exceeding the EAC, compared with Holy Loch 2009. Despite this reduced pollutant exposure, DIA, a proapoptotic gene, was up-regulated in both male and female fish from Holy Loch in 2010. A similar pattern was seen at Tancred, where moderate levels of contaminants and biological effect data are observed in these fish, with only one male showing elevated concentrations of plasma Vtg (0.354 µg mL^−1^) and DIA being up-regulated in both male and female fish. Despite this, induction of VIT and CHO gene expression was apparent in male fish from Tancred. In addition to this, Vtg has also been reported in male flatfish from the Gulf of Mexico and positively linked with polycyclic aromatic hydrocarbons and some metal exposure [47].

At St. Andrews, fish displayed moderate levels of contaminants and biomarker response, with male flounder demonstrating significant induction of VIT and CHO genes and DIA up-regulated in both male and female fish. GST was also slightly up-regulated (females more so than male), while MTT and ALD were suppressed in both sexes. Multivariate analysis showed St Andrews to have higher values for silver, cadmium, and arsenic compared with the other locations, which showed inconsistencies; there was a decrease in MTT, which is a metal-binding protein and has been shown to be induced by cadmium in flounder [48]. However, poor correlations between individual metals and MT induction in flounder have been previously reported by [49]. Metallothionein proteins are generally increased upon exposure to high levels of metal contaminants, which was not the case with this dataset. At Balcary Point, EROD activity in male fish and bile metabolites were below the BAC, but interestingly, the CYP1A gene was up-regulated. Female fish clustered on the basis of an increase in the mean in K, GSI, LSI, and EROD, with both GSI and LSI being the highest at Balcary compared with fish from other locations. Multivariate analysis showed Alloa fish to have lower values of lead compared with other sites, as well as less BDE66 and more BDE100 and BDE154. In male flounder from Alloa, VIT, CHO, and DIA were significantly up-regulated, while MTT and ALD were suppressed. In Irish flounder sampled from the Slaney, some genes were up-regulated (in particular c-Fos and DIA), but without contaminant and biomarker data from this location, it was difficult to make concrete conclusions on the cause-and-effect response to particular contaminants.

The limit of detection for plasma Vtg concentrations (<0.2 µgmL^−1^) was above the BAC available in flounder (0.13 µgmL^−1^), and with the majority of fish from most locations below the limit of detection, it was difficult to detect patterns in Vtg plasma concentrations and gene expression for VIT and CHO genes. Despite this, the induction of these genes in the fish exposures of E2 and lindane, coupled with induction in wild fish from HL09, Tancred, St. Andrews, and Alloa could potentially demonstrate a sensitive early-warning biological response to these types of contaminants, demonstrating acute and chronic exposure to potential endocrine-disrupting contaminants. The VIT and CHO genes could be incorporated as additional sensitive biomarkers in parallel with monitoring Vtg plasma levels in integrated monitoring programs. 

The DIA gene, being involved in apoptosis regulation, could also be a potential candidate for further exploration as a sensitive early-warning marker of exposure to contaminants. Interestingly, the CYP1A gene was induced in female fish and not male fish from HL09, perhaps demonstrating an impact on the phase I and II metabolism of the elevated PCBs (28, 52 and 118) in flounder at this site. The differences in gene expression between male and female fish indicate the need for the measurement of contaminants to be conducted in individual organisms in future monitoring programs.

### 4.3. Multivariate Analysis

Spatial differentiation was observed with contaminants in fish samples including metals, PCBs, and PBDEs. Alloa and Tancred clustered with lower values of lead than other sites, and Holy Loch clustered based on variation in PCBs, while Alloa, Holy Loch, and Bowling clustered due to differences in PBDEs. The same clustering and separation, however, was not apparent in the biological effects or gene expression data, which showed inconsistencies. Balcary Point separated from the other locations due to higher K, GSI, HSI and EROD, perhaps attributed to older fish sampled from this location. Also at Balcary Point, the MN response was 2–3-fold higher than in the other sites and 2-fold higher than the BAC. For gene expression data, Alloa was separated from other locations on LD2, showing increases in VIT, CHO, DIA and ALD genes, but this was inconsistent with actual gene expression data wherein Holy Loch demonstrated the highest induction of the VIT and CHO genes (with DIA and ALD genes driving the separation on LD2). In order to investigate relationships between gene expression profiles and higher-level biological effects (i.e., EROD, Bile, MN) and supporting parameters (GSI, LSI, K), regression analysis was conducted; for EROD, MN, and bile metabolites, it was found that none of the genes could predict the EROD response. Therefore, there was no relationship between EROD and any of the genes, potentially indicating a mismatch between the gene and later translation of the protein and indicating that the higher-organisational-level biomarkers potentially have more robust responses to moderate contaminant levels. This was not true for the Vtg and VTG and CHO genes, where these were shown to be more sensitive than Vtg in blood plasma in field samples.

There were inconsistencies between the higher biological effects’ responses and contaminant levels; for example, at Holy Loch, PCBs exceeded the EAC and the EROD fell below the EAC. Such inconsistencies have been noted previously and may be due to the inhibition of EROD activity by dioxin-like compounds [50], as inhibition usually only occurs at very high exposure to AhR-agonists with inverted U-shaped dose–response curves.

Contaminant levels and biological effects data suggested moderate contamination for most of the sites with the exception of Holy Loch in 2009, and at this site, the gene expression data showed significant changes. This could potentially indicate some sensitivity at the subcellular level of biological organisation at this site for some contaminants. It is apparent that acute exposure to single chemicals causes clear patterns in specific gene expression, whilst chronic exposure to mixed contaminants has very different effects on gene expression. There is evidence that long-term chronic exposure to contaminants, especially complex mixtures, may produce very different effects on gene expression than predictions would indicate from short-term acute exposure to single chemicals [17]. Despite the lack of clear cause-and-effect relationships between contaminants, higher biological effects’ responses and/or genes, it is clear from the multivariate analyses that there are differences between the sites for different levels of contaminants and biological effects, warranting that further assessment criteria be developed for other contaminants and biological effects in flounder. A limitation of the multivariate analysis was that not all biological effect data could be included due to missing data. This meant a much-decreased dataset for analysis. Also, if contaminant data were available for separate genders rather than mixed genders and in individual fish, this would strengthen future statistical analyses, which might allow for future novel associations to be made between contaminants and effects. This is further shown with the inter-gender differences in gene expression between male and female fish.

Nevertheless, the present study highlights and confirms diagnostic genes for use as biomarkers of exposure with which to model contaminants in particular VIT and CHO for exposure to E2. Other candidate genes were identified in the exposures, including c-FOS and ALD for PFOA and HEP and DIA for 3-MC. The ALD gene is involved in carnitine biosynthesis, which is required for fatty acid metabolism, a process that PFOA disrupts, while cFOS has many roles including responses to various stressors. CYP and DIA gene expression proved to be promising indicator genes with clear links to both elevated levels of PCBs as shown in the Starplots, and to induction of EROD activity and high levels of bile metabolites, thus indicating PAH exposure. An increase in DIA would sensitise cells to apoptotic signals, so it can be speculated that it is a response to chronic cellular damage, which increases the chances that the cell commits suicide before any neoplastic transformations can take place. GST up-regulation also showed promising indication of xenobiotic exposure, and visual relationships were observed with monitoring sites with higher levels of metals; previous studies have shown the link between MTT and cadmium. There is a clear link between VIT and CHO up-regulation in response to estrogenic compounds, and in this study, some sites showed elevated levels of the genes, whereas the plasma vitellogenin levels were below background levels. Therefore, we recommend these two complementary genes for potential use as an early response to estrogenic exposure in male flounder in combination with Vtg in monitoring programmes. The HEP gene is predominantly known as a gene which responds to bacterial infection and redirects iron to the liver, which is believed to inhibit bacterial growth in blood due to starvation of iron. However, it is also frequently found to be up-regulated in polluted environments that flounder inhabit, albeit to a much lesser extent than by infection. This could be a response used to minimise infection to which the animal might be more susceptible if impacted by pollutants and requires further exploration.

In the field monitoring study, there were no clear associations between the higher-organisational-level biomarkers and the genes, indicating that perhaps some of the protein synthesis steps had not yet taken place. However, with the quantitative scoring system used, there were associations made between contaminants and certain genes (i.e., Holy Loch PCBs and elevated CYP and VIT), allowing more of an insight into the data.

## 5. Conclusions

The VIT and its precursor CHO gene showed promise for use as early-warning biological responses in male wild-caught flounder as a technique to complement plasma Vtg levels in monitoring programmes. The Starplots demonstrated that the genes with a specific mode of action (e.g., oxidative stress and detoxification of metals) were not visibly clearly related to the metal levels, which were mostly at moderate levels in the fish liver samples; perhaps this link can only be seen with higher contamination levels. VIT and CHO genes were significantly increased in response to exposure to contaminants such as E2 and lindane and were moderately induced in all of the exposures; these genes have been recommended strongly as markers of exposure of male flounder to estrogenic contaminants but could potentially be responding to other contaminants, and further studies on mixture effects are warranted. Other candidate biomarker genes were identified in the exposures, including c-Fos for PFOA and HEP for lindane and 3-MC. However, there was little evidence to show that expression of these genes correlated with contaminant levels in wild-caught fish from Scotland and Ireland, and it might be specific to the particular contaminants. The use of multivariate analysis methods such as LDA and PCA, as well as regression analysis, can discriminate between fish from different sites based on a range of measures and is recommended for future use on monitoring data in order to investigate the associations and relationships between confounding factors such as age when assessing biological effects and contaminant data. The use of mixed-gender tissue pools for contaminant analysis reduced the power to identify associations with genes and specific biological responses. It is therefore recommended that fish are separated by gender upon initial sampling during monitoring and that contaminant analysis is carried out on individuals. In wild-caught fish, there are genes (CFOS, HEP and DIA) which might be better indicators of chronic exposure, although they are not clearly linked to specific contaminants (only general contamination) but could be used as supporting parameters. When extrapolating with the wild-caught fish, the genes CYP, DIA, and GST showed clear links to PCB and PAH exposure in the fish and could be used with VIT and CHO-L as a clear cause-and-effect marker with regard to MSFD objectives. The full qPCR array was not validated for future use as a new and innovative method with direct cause-and-effect establishment between gene- and system-level responses but could be used as a supporting tool for monitoring. Further work is warranted to investigate links between gene expression and liver pathology.

## Figures and Tables

**Table 1:** Genes and functions chosen for this study.

Acronym	Gene	Function
ALD	Aldehyde dehydrogenase 9A1	Xenobiotic metabolism, oxidises amino aldehydes
UGT	UDP-glucuronosyltransferase 1B	Xenobiotic metabolism, detoxification of organics
SOD	Cu/Zn superoxide dismutase	Xenobiotic metabolism, detoxification of superoxide radicals
GST	Glutathione S-transferase rho class	Xenobiotic metabolism, detoxification of organics
MTT	Metallothionein	Xenobiotic metabolism, toxic metal defense
CYP1A	Cytochrome P4501A	Xenobiotic metabolism, metabolises polyaromatic hydrocarbons
CFOS	C-fos	Stress response, multifunctional transcription factor
HEP	Hepcidin	Stress response, regulates iron distribution
VIT	Vitellogenin	Endocrine disruption, egg protein induced in male fish by estrogens
CHO	Choriogenin L	Endocrine disruption, egg protein induced in male fish by estrogens
DIA	Diablo1	Apoptosis regulator, increases sensitivity to apototic signals
GCBP	Cytosolic non-specific dipeptidase	Glutathione synthesis but function uncertain
	Elongation factor alpha	Reference gene
	Alpha tubulin	Reference gene

**Table 2:** Assessment criteria as detailed in ICES (2012) [10] for biological effect monitoring and available assessment criteria for chemical determinants in fish, OSPAR, 2008/2009 [36]. Fish (µgkg^−1^ wet weight), except EAC^passive^ lipid weight, and EC^a^ max food limit. X denotes not available.

	<BAC	<EAC^passive^	<EC^a^	
Metals				
Cd	26	X	X	
Hg	35	X	500 ^(a)^	
Pb	26	X	X	
PCBs				
CB 28	0.1	64	X	
CB52	0.08	108	X	
CB101	0.08	120	X	
CB118	0.1	24	X	
CB138	0.09	316	X	
CB153	0.1	1600	X	
CB180	0.11	480	X	
Biomarkers	Species	Sex	<BAC	<EAC
EROD (pmol min^−1^ mg protein^−1^)	Flounder	M	24	X
PAH bile metabolites (pyrene type ng mL^−1^); synchronous scan fluorescence	Flounder	M&F	16	X
VTG plasma (μg mL^−1^)	Flounder	M	0.13	X
Micronuclei frequency in erythrocytes	Flounder	M&F	0–0.3	X

^a^ Caution should be taken here, as this value is a seafood maximum limit rather than a value linked to toxicological response in fish (Regulation 1881/2006) [39].

**Table 3:** Fish metal (mg kg^−1^ wet weight) and PCB (µg kg^−1^ lipid) liver concentrations in flounder (mean ± 95% confidence interval). Assessments are based on estimating the 95th percentile of lognormal distribution based on sample median and standard deviation and classified as per [36]: blue (<BAC), green (>BAC <EAC) and red (>EAC). ND: below limit of detection; TR: below limit of quantification.

	Balcary Point	Holy Loch 2009	Alloa	St. Andrews	Tancred	Holy Loch 2010	Bowling
**Cd**	307.9 ± 81.9	57.6 ± 8	107.6 ± 10	236.8 ± 107	95.8 ± 12.5	76.5 ± 42.7	53.3 ± 6.5
**Hg**	102.9 ± 24.1	26.3 ± 3.3	114.6 ± 20.9	61.6 ± 86.1	121.5 ± 23.6	42.8 ± 15	57.4 ± 9.9
**Pb**			46.8 ± 27.4	31.3 ± 18	61.8 ± 5.3	140 ± 28.5	150 ± 50.3
**CB28**	ND	49.5 ± 23.5	3.4 ± 2	0.1 ± 0.9	2.7 ± 0.9	13 ± 8.9	7.3 ± 2.5
**CB52**	ND	78.6 ± 44.2	4.4 ± 2.7	0.1 ± 1.1	5.8 ± 2	24.8 ± 15.3	12.7 ± 3.8
**CB101**	TR	77.3 ± 39.4	9.2 ± 5	0.5 ± 2.9	10.7 ± 4.7	32.2 ± 9.4	5.8 ± 1.5
**CB118**	TR	63.9 ± 35.1	7.2 ± 3.7	5.4 ± 1.9	8.2 ± 3.6	32.5 ± 7.7	8.3 ± 2.1
**CB138**	TR	59 ± 29.8	13.3 ± 7.6	5.8 ± 3.7	16.9 ± 7.1	45.9 ± 12.9	10.8 ± 2
**CB153**	103 ± 4.2	122.8 ± 64.2	22.5 ± 10.1	7 ± 4.2	24.9 ± 12	75.9 ± 20	15.2 ± 2.6
**CB180**	TR	66.1 ± 33.4	21.8 ± 13	3.8 ± 4.3	19 ± 9.6	43 ± 20.5	11.4 ± 3

**Table 4:** Biomarker data (Median ± SD) including hepatic EROD (pmol ^−1^min ^−1^mg ^−1^ protein S9), bile metabolite concentrations (ng mL^−1^) SFS method (1-OH pyrene equivalents), MN (MN/1000 erythrocytes; ‰) and plasma vitellogenin (μg mL ^−1^) in flounder from seven Scottish locations including Balcary point, Holy Loch 2009 (HL09) and 2010 (HL10), St Andrews, Tancred, and Bowling. Assessments are based on estimating the 95th percentile of lognormal distribution based on sample median and standard deviation and compared to criteria as per ICES (2012) [10] (i.e., response <BAC (blue) and >BAC <EAC (green), >EAC (red)).

	Gender	Balcary Pt.	HL09	Alloa	St. Andrews	Tancred	HL10	Bowling
**Condition index (K)**	Mixed	1.19 ± 0.07	1.24 ± 0.05	1.01 ± 0.05	1.03 ± 0.05	1.07 ± 0.04	1.15 ± 0.08	1.07 ± 0.04
**Gonadal somatic index (GSI)**	Mixed	7.37 ± 1.94	1.79 ± 0.7	0.45 ± 0.36	1.35 ± 0.38	0.82 ± 0.27	1.52 ± 0.51	0.9 ± 0.37
**Liver somatic index (LSI)**	Mixed	1.99 ± 0.31	1.59 ± 0.2	1.19 ± 0.08	1.46 ± 0.18	1.0 ± 0.09	1.45 ± 0.21	1.3 ± 0.15
**EROD**	Male	16.22 ± 25.92	42.17 ± 18.3	3 ± 1.17	35.7 ± 9.7	13.5 ± 6.7	77.35 ± 33.9	66.2 ± 13.1
	Female	3 ± 0	13.2 ± 7.72	3	18 ± 25.18	12.9 ± 12.3	52 ± 30.3	48.5 ± 31.4
**Bile metabolites**	Mixed	247.7 ± 58.4	857.6 ± 1108	1201 ± 418	159.72 ± 28.1	2163.3 ± 2066.8	1055.3 ± 1036.2	1614.4 ± 464
**Vitellogenin (Vtg)**	Male	none	none	0.2 ± 1.45 × 10^−17^	0.2 ± 1.4 × 10^−17^	0.2 ± 0.04	0.2 ± 11.4	0.2 ± 0
**Micronucleus (MN)**	Mixed	0.6 ± 0.4	0.4 ± 0.27	0.3 ± 0.2	0.3 ± 0.22	0.2 ± 0.13	0.2 ± 0.23	0.2 ± 0.36

## Data Availability

The original data presented in the study are included in the article/Supplementary Materials; further inquiries about the data can be directed to the corresponding author.

## Supplementary Materials

The following supporting information can be downloaded at: https://www.mdpi.com/article/10.3390/toxics13030203/s1, Table S1: Primer pairs and database information; Figure S1a–g: Gene expression in wild caught flounder. Figure S2a: LDA of site differences with metal measurements; Figure S2b: LDA of site differences and PCB measurements; Figure S2c: LDA of site differences with PBDE congener concentrations.
