# Supplementary material for: Investigating the Use of Diagnostic Genes in Integrated Monitoring with a Laboratory and Field Study on Flounder (Platichthys flesus)
Source: Toxics. 2025 Mar 12;13(3):203. doi: 10.3390/toxics13030203 (PMC11945703; doi:10.3390/toxics13030203)
Supplement: Supplementary file 1 [file toxics-13-00203-s001.zip › toxics-3407761-supplementary.pdf]

Supplementary material

Table S1. Primer pairs and database information

Gene	Primer sequence
VIT_F	CTG ACC TTC GTG GAT ATT GAG
VIT_R	ATC TGA GCC TCG GCA TTG
ALPHATUBULIN_R	AGA TGA CAG GGG CAT AGG TG
ALPHATUBULIN_F	CAC AGC CTC ACT TCG TTT TG
ELF1ALFFA_R	CGA GCA TAC CGG GCT TAA TG
ELF1ALFA_F	CTT GAG CGC AAG GAG GCT AA
SOD_R	CAT TGA GGG TGA GCA TCT TG
SOD_F	TGG AGA CAA CAC AAA CGG G
CYP1A_R	CTC ATC ACT GAG GGT CAC CA
CYP1A_F	CTG GAG GAA CAC ATC TGC AA
GLUTHATHIONE-R_R	CGA GGT TGC TAC GAT AAA TGC
GLUTHATHIONE-R_F	GGC TTC TTG CGT GAA TGT TGG
GLUTHATHIONE-S_R	GAA AAC CGA CAC ATC AGC CA
GLUTHATHIONE-S_F	TCA CGC TCA CCC AGA AAA TG
HEPCIDIN_R	ATG CTC AGC AGC TGC ATT GT
HEPCIDIN_F	TCA CCA GCA GAG TCA AAG AAC T
UGT1B_R	AGA GAG CCC CAT GAC TGA GA
UGT1B_F	CCT TCC CGC AGA GAG TCA TA
C-FOS_R	GGG TTA AGA CAA AGC ACG TCA
C-FOS_F	TGC CTA TAA CAC CGA TGT AGC A
ALDEHYDE_R	GAG CAG GAG CAG ACT TCC AC
ALDEHYDE_F	GGG AGA AGA TTG CAA AGC TG
METALLOTHIONEIN_R	AGC CGA ATG GGC AGC ATG G
METALLOTHIONEIN_F	CTG CGA ATG CTC CAA GAC T
GLUTAMATE_R	GGA CGT CGC TTA ACA ATG CT
GLUTAMATE_F	CGA CAT GGA TCC TGA TCT GA
CHORIOGENIN_R	GTG GCA GGG CAT TGA GTT AC
CHORIOGENIN_F	CCT CCC AGA AGT CCA GTG AA
DIABLO_R	ACT GGG CCA CCT GAA TGT TT
DIABLO_F	CCT CAT TCG AGG AGG ATT CG

[a]

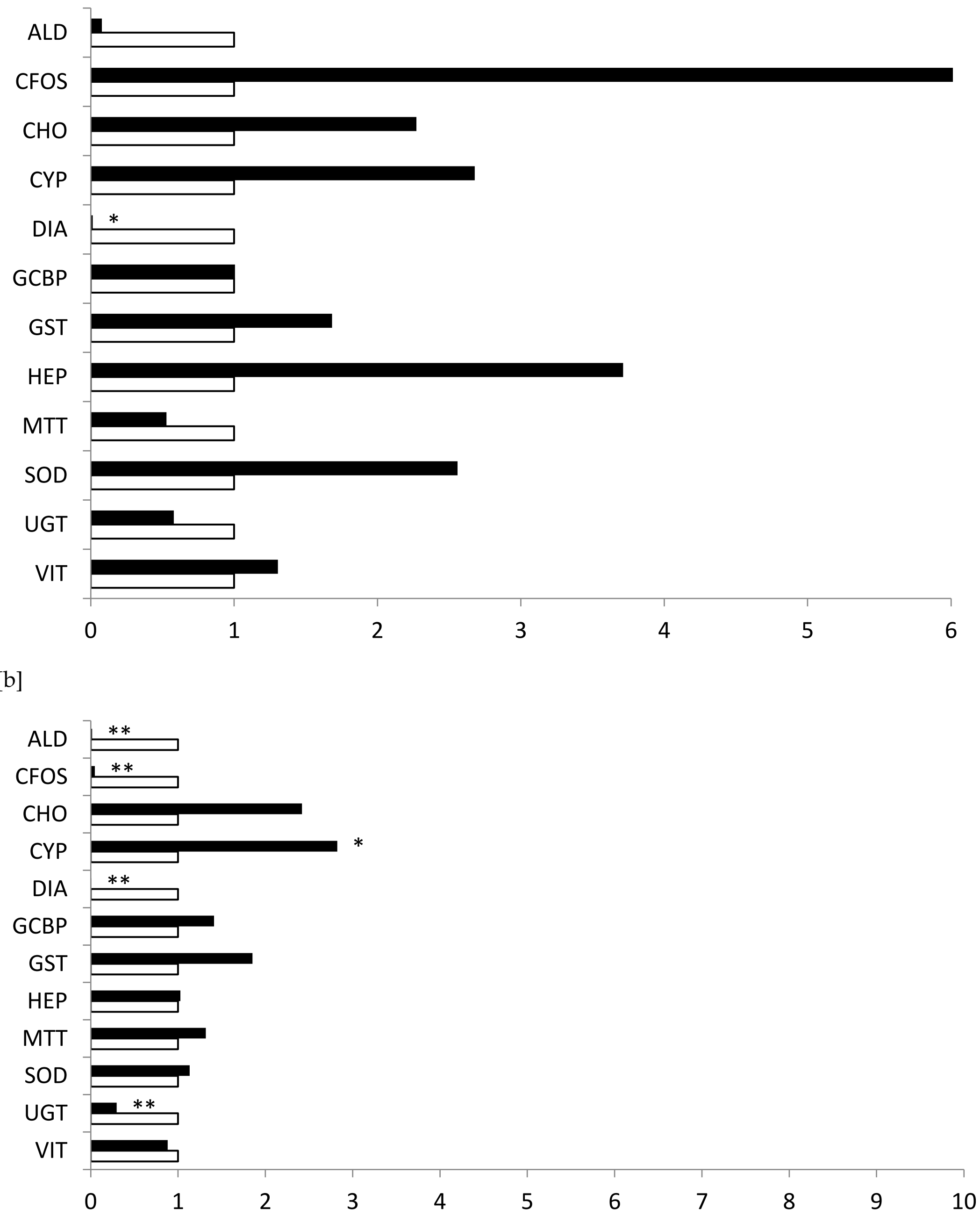

Figure S1a. Gene expression in wild caught flounder from Balcary Point [■] in [a] male, [b] female flounder with significant differences to unexposed saline control [□] indicated (p< 0.001=***, p<0.01=**, p<0.05=*).

[a]

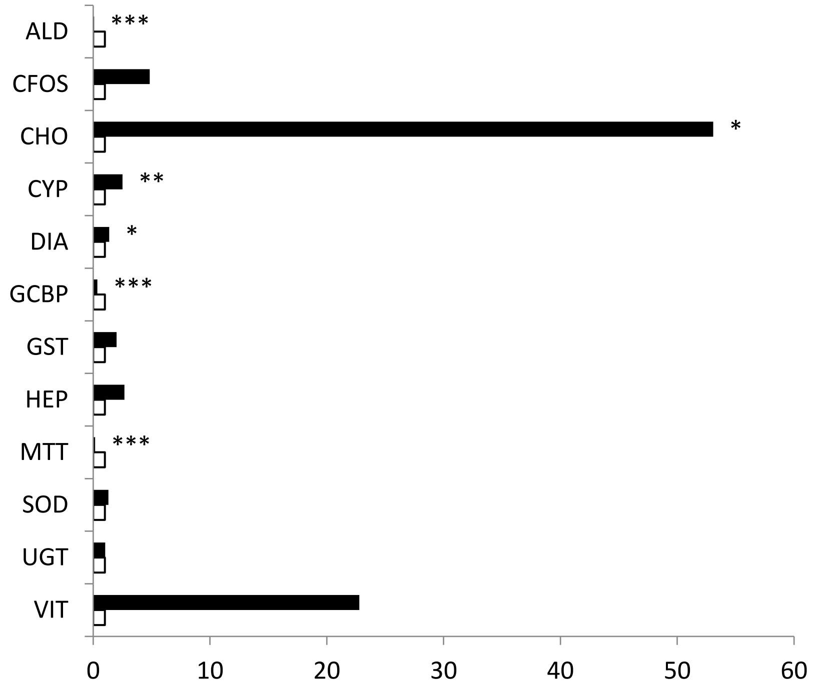

[b]

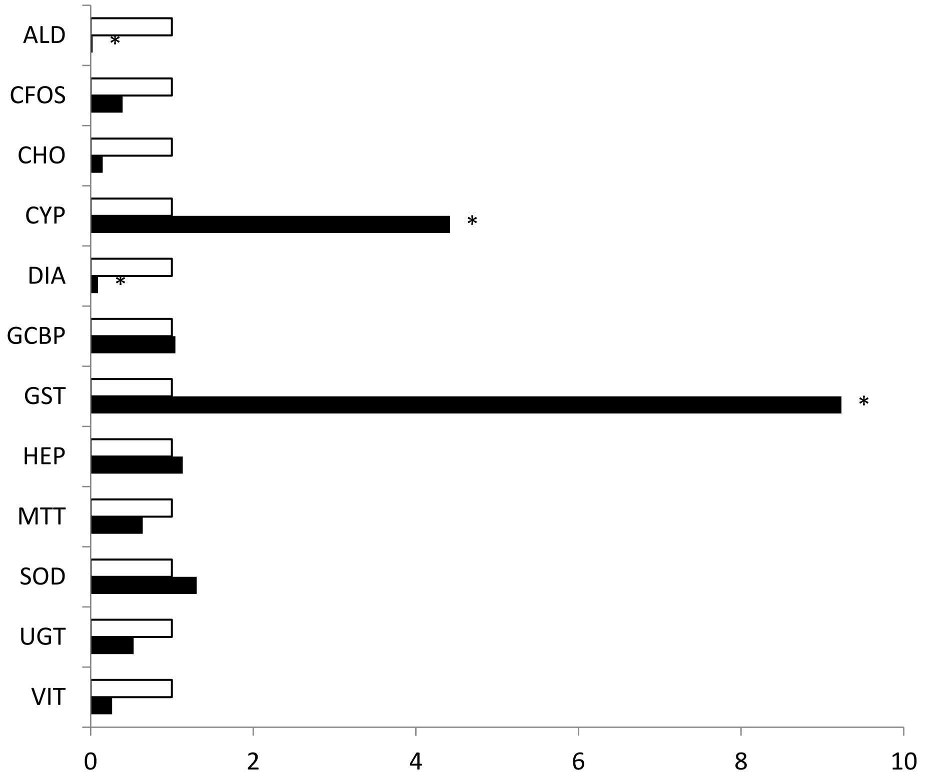

Figure S1b. Gene expression in wild caught flounder from the Holy Loch 1 [■] (2009) (Inner Firth of Clyde) in [a] male, [b] female flounder with significant differences to unexposed saline control [□] indicated (p< 0.001=***, p<0.01=**, p<0.05=*).
[a]

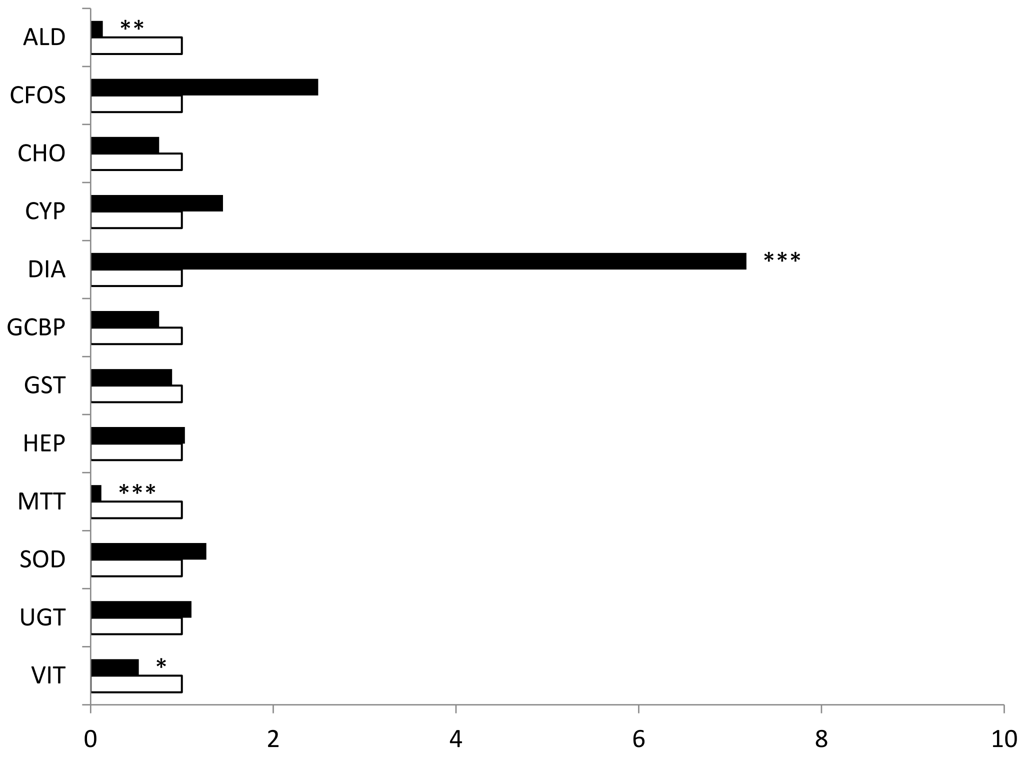

[b]

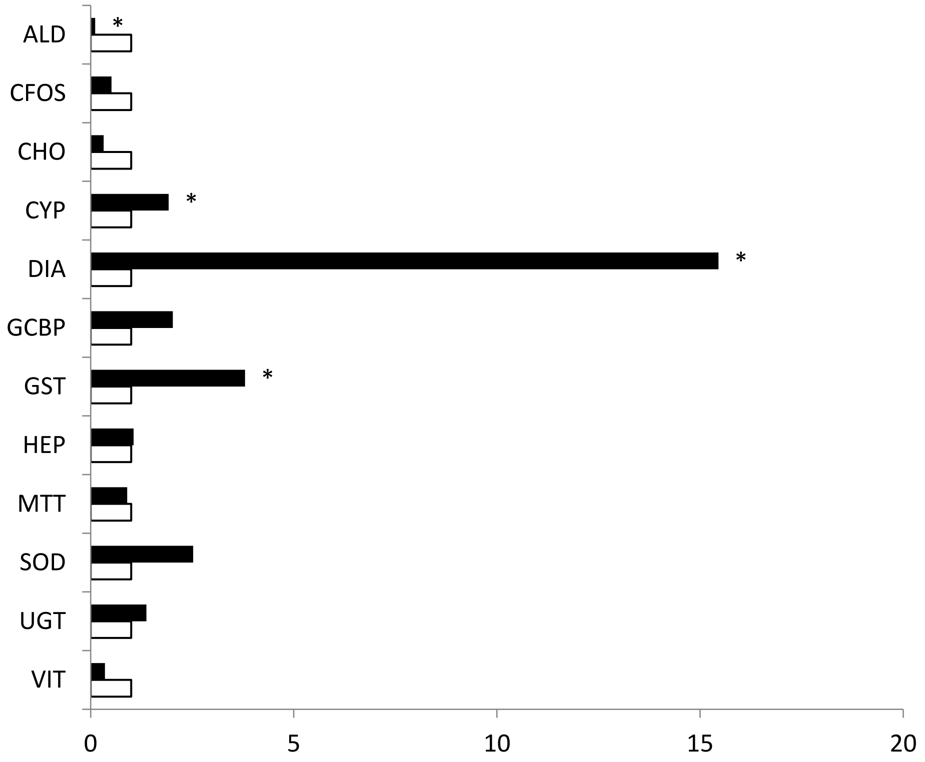

Figure S1c. Gene expression in wild caught flounder from the Holy Loch 2 [■] (2010) (Inner Firth of Clyde) in [a] male, [b] female flounder with significant differences to unexposed saline control [□] indicated (p< 0.001=***, p<0.01=**, p<0.05=*).

[a]

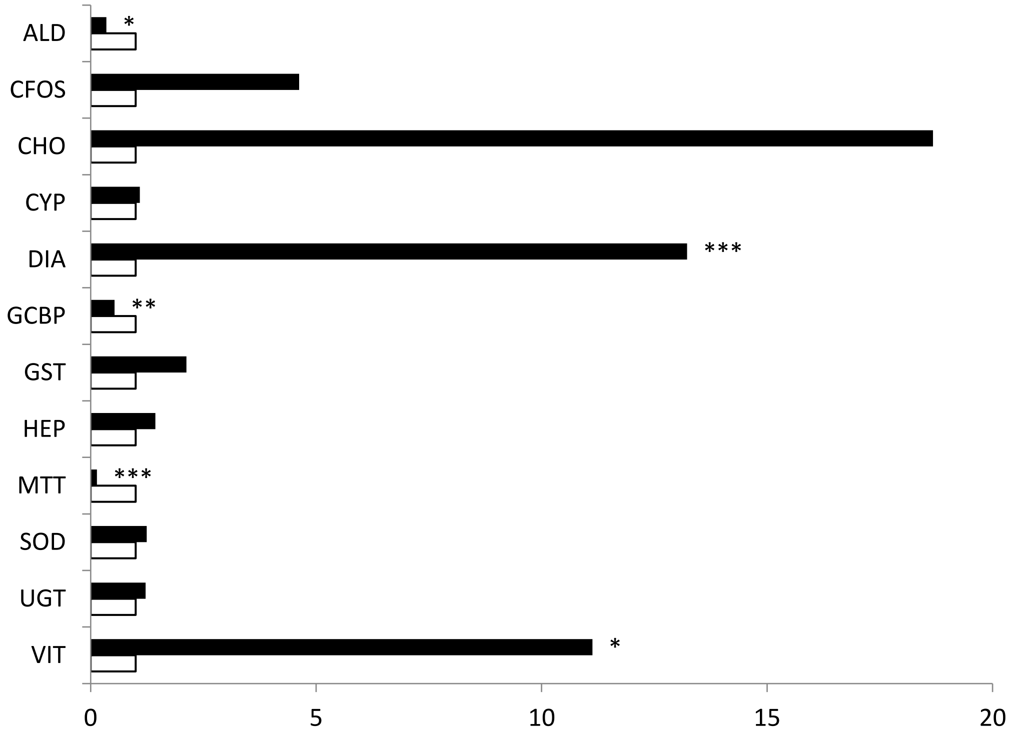

[b]

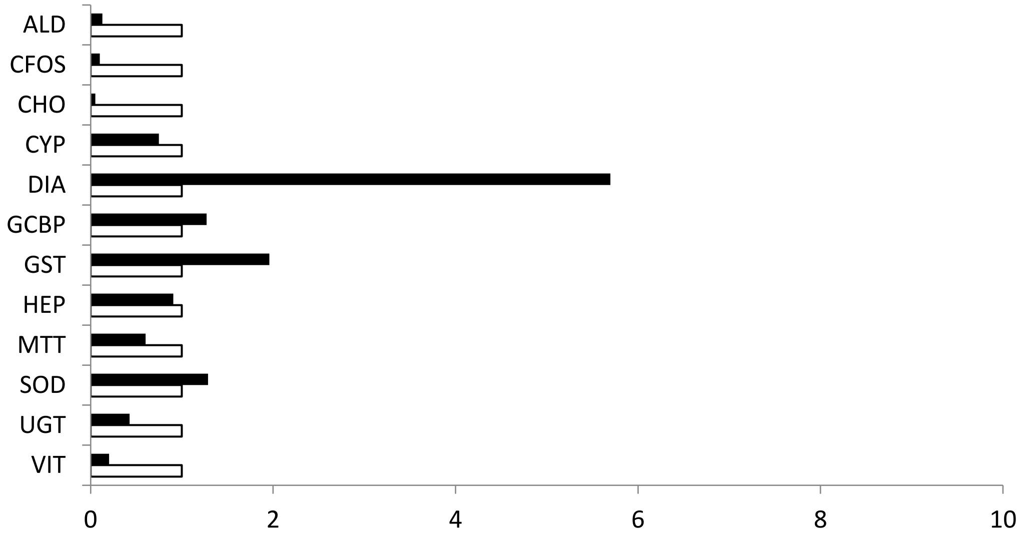

Figure S1d. Gene expression in wild caught flounder from the St. Andrews [■] in [a] male, [b] female flounder with significant differences to unexposed control [□] indicated (p< 0.001=***, p<0.01=**, p<0.05=*).

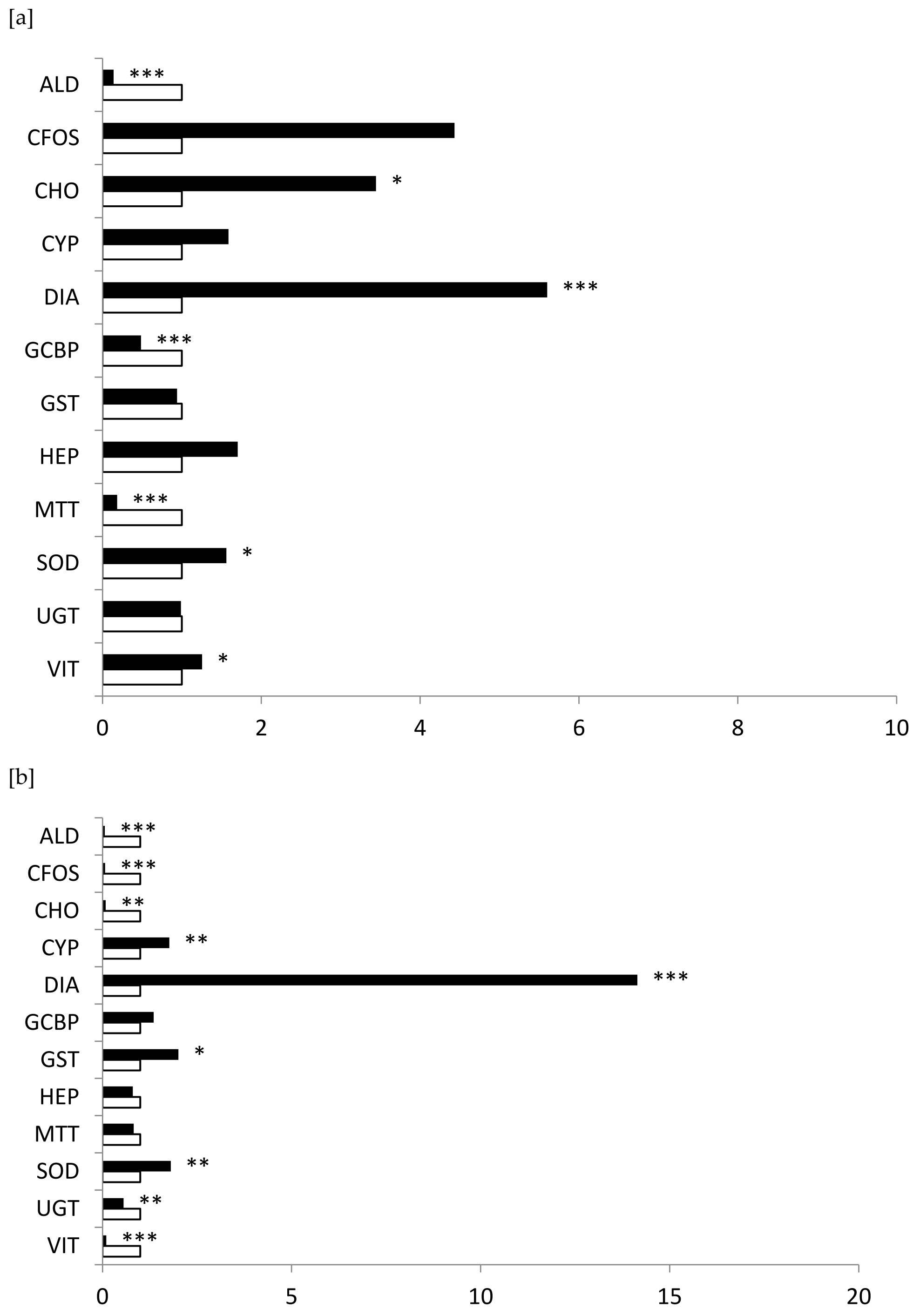

Figure S1e. Gene expression in wild caught flounder from the Tancred [■] in [a] male, [b] female flounder with significant differences to unexposed control [□] indicated (p< 0.001=***, p<0.01=**, p<0.05=*).

[a]

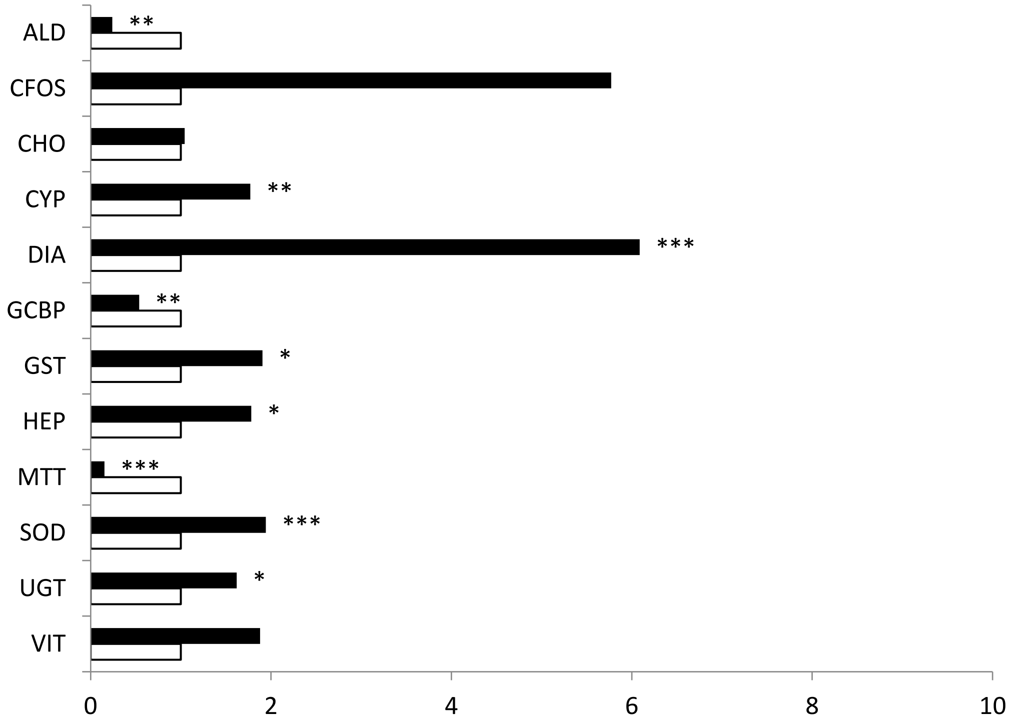

[b]

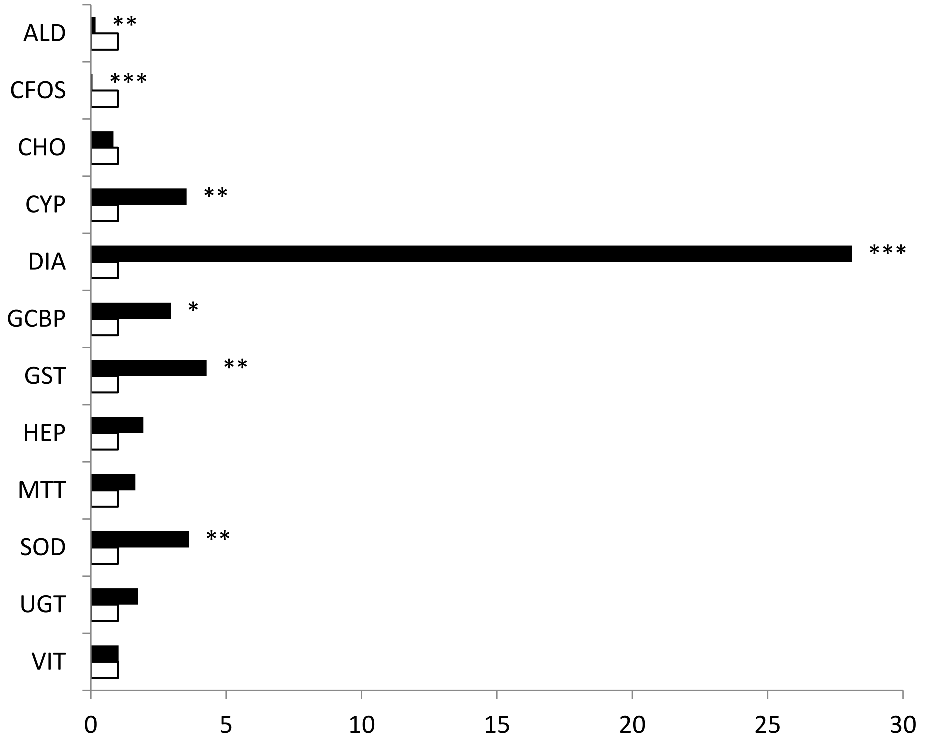

Figure S1f. Gene expression in wild caught flounder from the Bowling [■] in [a] male, [b] female flounder with significant differences to unexposed saline control [□] indicated ($p < 0.001$=***, $p<0.01$=**, $p<0.05$=*).

[a]

[b]

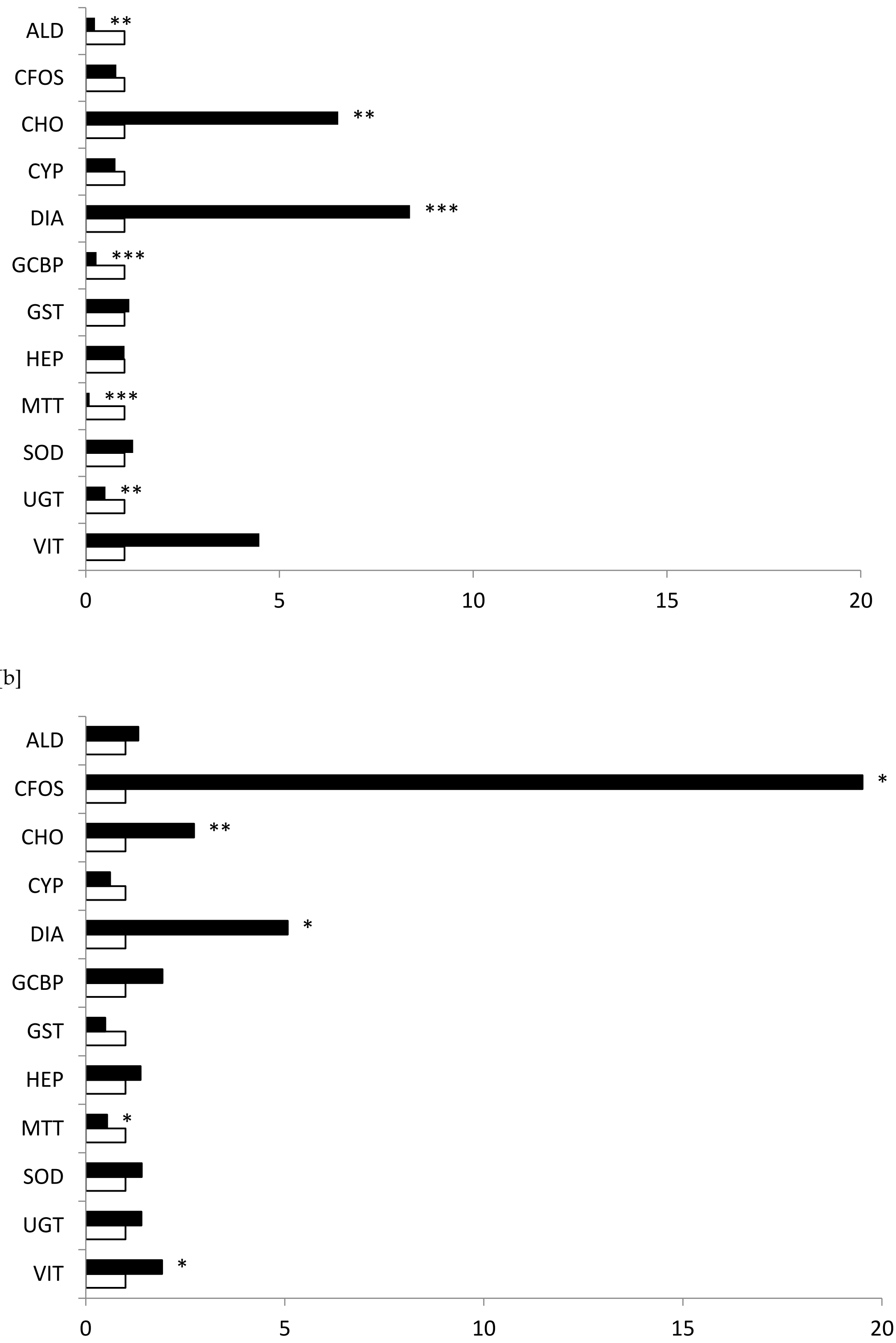

Figure S1g. Gene expression in male wild caught flounder from [a] Alloa[■] [b] Slaney [■] with significant differences to unexposed saline control [□] indicated (p< 0.001=***, p<0.01=**, p<0.05=*).

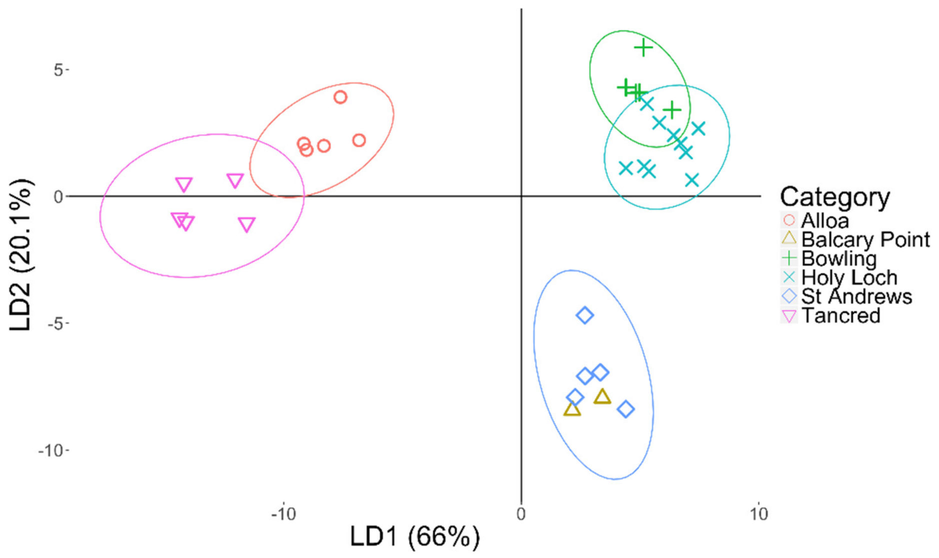

Figure S2a. LDA of site differences with metal measurements.

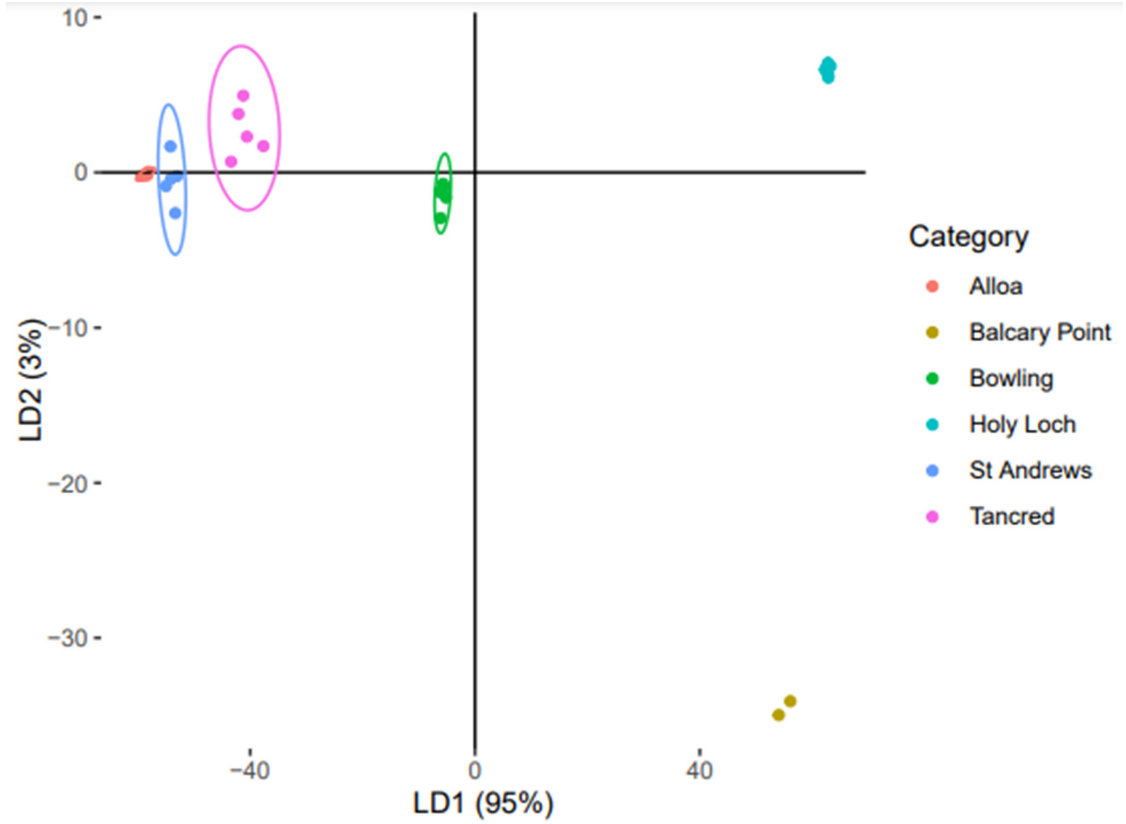

Figure S2b. LDA of site differences and PCB measurements.

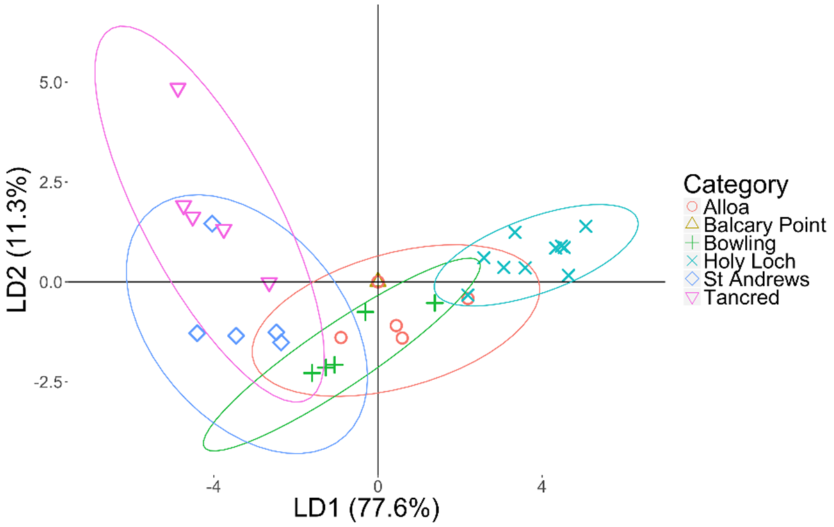

Figure S2c. LDA of site differences with PBDE congener concentrations.
